# Synthesis and Cytotoxicity Evaluation of Denitroaristolochic Acids: Structural Insights and Mechanistic Implications in Nephrotoxicity

**DOI:** 10.3390/biom15071014

**Published:** 2025-07-14

**Authors:** Jianfei Gao, Mengtong Zhao, Jianhua Su, Yi Gao, Xiaofeng Zhang, Yongzhao Ding, Xiaoping Liu, Yang Luan, Chun Hu

**Affiliations:** 1 Key Laboratory of Structure-Based Drug Design & Discovery (Ministry of Education), Shenyang Pharmaceutical University, Shenyang 110016, China; gaojianfei1996@163.com (J.G.); zmt18240302933@163.com (M.Z.); 18522280724@163.com (J.S.); yigao2022@163.com (Y.G.); z1121628369@126.com (X.Z.); iyuzuru@126.com (Y.D.); xiaopingliu@syphu.edu.cn (X.L.); 2 School of Public Health, Hongqiao International Institute of Medicine, Shanghai Jiao Tong University School of Medicine, Shanghai 200025, China

**Keywords:** denitroaristolochic acids, total synthesis, Suzuki-Miyaura coupling, Friedel-Crafts cyclization, nephrotoxicity, structure-activity relationship

## Abstract

The efficient synthetic routes and evaluates cytotoxic profiles of denitroaristolochic acids II–V (DAA-II–V) were demonstrated in this study. Based on retrosynthetic analysis, a modular synthetic strategy was developed through Suzuki–Miyaura coupling, Wittig reaction, and bismuth triflate-catalyzed intramolecular Friedel–Crafts cyclization to efficiently construct the phenanthrene core. Process optimization significantly improved yields: aryl bromide intermediate A reached 50.8% yield via bromination refinement, while arylboronic ester intermediate B overcame selectivity limitations. Combining Darzens condensation with Wittig reaction enhanced throughput, achieving 88.4% yield in the key cyclization. Structures were confirmed by NMR and mass spectra. CCK-8 cytotoxicity assays in human renal proximal tubular epithelial cells revealed distinct toxicological profiles: DAA-III and DAA-IV exhibited IC_50_ values of 371 μM and 515 μM, respectively, significantly higher than the nitro-containing prototype AA-I (270 μM), indicating that the absence of nitro group attenuates but does not eliminate toxicity, potentially via altered metabolic activation. DAA-II and DAA-V showed no detectable cytotoxicity within assay limits, suggesting reduced toxicological impact. Structure–activity analysis exhibited that the nitro group is not essential for cytotoxicity, with methoxy substituents exerting limited influence on potency. This challenges the conventional DNA adduct-dependent toxicity paradigm, implying alternative mechanisms like oxidative stress or mitochondrial dysfunction may mediate damage in denitro derivatives. These systematic findings provide new perspectives for AA analog research and a foundation for the rational use and safety assessment of *Aristolochiaceae* plants.

## 1. Introduction

The historical medicinal applications and evolving toxicity awareness of *Aristolochiaceae* plants reflect cross-regional civilization interweaving characteristics [1]. Documented utilization of these plants in global traditional medical systems dates back to the second century BC, with diversified therapeutic implementations across major ancient civilizations. In Chinese medical classics *Shennong Bencaojing* (ca. 200 BC), *Aristolochia* species were prescribed for cough and asthma relief while *Asarum* species served as essential analgesics in clinical practice [2]. Concurrently, Roman physicians employed *Aristolochia* clematitis for gynecological treatments [3], and Indigenous South American communities developed antiophidic preparations from *Aristolochia* rotunda [4,5].

The Age of Exploration (16th century) facilitated transcontinental knowledge exchange regarding these botanicals [6]. Notably, the Dutch East India Company introduced Indian *Aristolochia* species as alternative antimalarial agents to European markets, paralleling Li Shizhen’s systematic documentation in *Bencao Gangmu* (1596 AD) [7,8,9,10]. This seminal pharmacopeia not only standardized detoxification processing techniques but also established pregnancy contraindications. Notably, traditional medical records predominantly provided empirical warnings through terms like “cautious use” and “prolonged administration prohibition”, without elucidating the underlying toxicological mechanisms through scientific investigation.

Aristolochic acids (AAs) are naturally occurring components widely distributed in plants of the *Aristolochia* and *Asarum* genera within the *Aristolochiaceae* family, and about 20 structural analogs have been identified (Table 1). Although the AAs family comprises numerous members, significant toxicity variations exist among different structural analogs, with some demonstrating negligible toxicity. Research has revealed that AA-I exhibits the most potent toxicity profile, demonstrating nephrotoxicity, mutagenicity, and carcinogenicity [11]. The advancement of modern toxicological understanding of AAs originated from a pivotal discovery in 1964 during the 1960s to 1980s, when Romanian researchers first documented the induction of renal tubular lesions by these compounds [12]. However, restricted scientific communication between Eastern and Western blocs impeded international recognition of these findings. This scientific paradigm shifted significantly in 1991 following a public health crisis: the accidental administration of *Aristolochia* fangchi-containing herbal preparations at a Belgian weight-loss clinic resulted in 107 cases of aristolochic acid nephropathy, with 19 patients subsequently developing urothelial carcinoma [13]. This discovery drew medical attention to an endemic nephropathy prevalent in the Danube River basin (particularly in Croatia), a progressive disease characterized by renal fibrosis that affected large populations in the region, with approximately half of the cases developing concurrent urothelial malignancies [14].

This epidemiological event catalyzed global scientific reevaluation of AA toxicity. Mechanistic studies revealed that AA metabolites form persistent DNA adducts, inducing characteristic from A:T to T:A transversion mutations in TP53 tumor suppressor gene [15]. Notably, the identified mutational hotspots (codon 131, 179, and intron 6 splice site) exhibited distinct patterns from those associated with tobacco smoking or analgesic-induced carcinogenesis [16]. These molecular insights prompted the International Agency for Research on Cancer (IARC) to classify AAs and their botanical sources as Group 1 carcinogens in 2002, establishing a milestone in toxicological risk assessment by integrating molecular biological evidence into traditional pharmacovigilance frameworks [17,18].

Phylogenetic analyses reveal that evolutionary processes and organ differentiation exert dual regulatory mechanisms on the biosynthesis of AAs from a plant metabolic perspective. Comparative metabolomics demonstrated distinct accumulation patterns among related species: *Aristolochia* species exhibited characteristic hyperaccumulation with AA-I concentrations reaching 773–1770 μg/g dry weight in fruits, whereas *Asarum* species showed significantly lower levels (ND-12 μg/g) in rhizomes. Notably, the AA content in *Aristolochia contorta* fruits (7–36 μg/g), though substantially reduced compared to hyperaccumulating congeners, still maintained two orders of magnitude higher concentrations than those detected in *Asarum* rhizomes [19,20,21,22,23,24,25].

The isolation and characterization of AA derivatives achieved significant progress in 1984 when Basudeb Achari’s research group4 first isolated denitroaristolochic acid IV (DAA-IV) from *Aristolochia indica* [26], providing conclusive evidence for the natural occurrence of this class of compounds. Subsequent technological advancements enabled Duan et al. to isolate DAA-II from *Aristolochia manshuriensis* in 2019 through optimized HPLC protocols [27], achieving a remarkable yield of 3.08%. However, this isolation was not accompanied by systematic bioactivity assessments, resulting in insufficient characterization of the analog’s pharmacological and toxicological profiles.

The current research bottleneck primarily lies in the unconfirmed natural occurrence of DAA-III and DAA-V, a phenomenon potentially attributable to multiple technical constraints. Firstly, substantial disparities in natural abundance are observed, with AA-III content in *Aristolochia* fangchi (0.05–0.1%) representing merely one-quarter of AA-I concentration. Secondly, conventional analytical techniques demonstrate insufficient sensitivity for AA-V detection, given its natural abundance frequently falls below standard quantification limits (<0.01%). Furthermore, complex phytochemical matrices significantly compromise the identification accuracy of low-abundance compounds. Although previous investigations have revealed marked differences in median lethal dose (LD_50_) and pharmacological profiles among AA subtypes, systematic elucidation of structure–activity relationships remains hindered by limited sample accessibility and methodological constraints in detection [28,29].

In summary, the scalable synthesis of high-purity denitroaristolochic acid II, III, IV, and V monomers through chemical synthesis approaches, combined with the establishment of standardized evaluation protocols for both toxicological properties (including acute/chronic toxicity and genotoxicity) and pharmacological activities (such as anti-inflammatory, immunomodulatory, and antitumor effects), presents a promising solution to address the current limitations of natural abundance and data fragmentation in phytochemical research. This integrated strategy enables the systematic elucidation of structure–activity relationships among aristolochic acid analogs while providing critical scientific foundations for three key applications: toxicity early-warning systems in *Aristolochiaceae* plants, medicinal potential exploration, and quality control standardization development. The proposed methodology demonstrates significant theoretical value and practical applicability in natural product research.

## 2. Materials and Methods

### 2.1. Chemical Synthesis

All common chemical reagents were obtained from Sinopharm Chemical Reagent Co., Ltd. (Shanghai, China), while specialty reagents were procured from commercial suppliers including Adamas-beta^®^. Anhydrous solvents were pretreated through standard purification and drying procedures prior to use. Column chromatography was performed using 200–300 mesh silica gel (Qingdao Marine Chemical Factory, Qingdao, China), and thin layer chromatography (TLC) analyses were conducted on GF254-precoated plates (Yantai Chemical Industry Research Institute, Yantai, China). The liquid chromatography–mass spectrometry (LC-MS) analyses were performed on an Agilent 1100 series LC-MS system equipped with an electrospray ionization (ESI) source. Nuclear magnetic resonance (NMR) spectra were recorded at 25 °C using Bruker AVII-500 MHz and 600 MHz spectrometers (Karlsruhe, Germany). The ^1^H and ^13^C NMR chemical shifts were referenced to tetramethylsilane (TMS) or residual solvent peaks as internal standards with perdeuterated dimethyl sulfoxide (DMSO-*d*_6_) as the solvent. High-resolution mass spectrometry (HRMS) data were acquired in ESI mode using an Agilent Technologies LC/MSD TOF system (Agilent Corporation, Santa Clara, CA, USA) with direct sample infusion.

The chemical names, chemical structures, and CAS registry numbers of target compounds and key intermediates are listed as Appendix A.

#### 2.1.1. Synthesis of 3-Bromo-4,5-dihydroxybenzaldehyde (**2**)

A solution of 3,4-dihydroxybenzaldehyde (10.00 g, 72.40 mmol) in anhydrous methanol (120 mL) was prepared under nitrogen atmosphere and cooled to 0 °C using an ice-salt bath. Liquid bromine (Br_2_, 13.88 g, 86.88 mmol) was added dropwise via a pressure-equalizing dropping funnel at a controlled rate of 0.5 mL/min, maintaining temperature fluctuations within ±2 °C throughout the addition. Following the completion of bromine addition, the reaction mixture was gradually warmed to 30 °C at a controlled rate of 2 °C/min and stirred continuously for 6 h. Reaction progress was monitored by TLC (eluent: petroleum ether/ethyl acetate = 3:1, *v*/*v*) until complete consumption of the starting materials was observed. The resulting mixture was quenched by slow addition to ice-cold water (200 mL, 0 °C), inducing precipitation of light yellow microcrystals. The crude product was collected by vacuum filtration through a Büchner funnel, followed by sequential washing with ice-cold water (three 30 mL portions) and chilled methanol (two 10 mL portions) to remove residual hydrogen bromide and unreacted bromine. Subsequent drying afforded white needle-like crystals (11.00 g, 70.1% yield). M.p.: 224.5–226.1 °C. ^1^H NMR (600 MHz, DMSO-*d*_6_) δ 9.69 (s, 1H), 7.56 (d, *J* = 1.9 Hz, 1H), 7.24 (d, *J* = 1.7 Hz, 1H). HRMS (ESI) *m*/*z*: 216.9487 ([M + H]^+^) (calcd for C_7_H_5_BrO_3_: 216.9501).

#### 2.1.2. Synthesis of 3-Bromo-4,5-methylenedioxybenzaldehyde (**3**)

The synthesis of the title compound was conducted by sequentially adding 3-bromo-4,5-dihydroxybenzaldehyde (11.00 g, 50.69 mmol), dibromomethane (CH_2_Br_2_, 13.22 g, 76.03 mmol), and anhydrous potassium carbonate (K_2_CO_3_, 35.03 g, 253.44 mmol) into a dried 500 mL three-necked flask. The reaction system was then charged with anhydrous *N*,*N*-dimethylformamide (DMF, 150 mL) under nitrogen atmosphere. The mixture was maintained at 110 °C with constant temperature stirring for 5 h. Reaction progress was monitored by TLC using dichloromethane (DCM)/methanol (30:1, *v*/*v*) as eluent until complete disappearance of the starting material spot. After cooling to ambient temperature (25 °C), the reaction mixture was carefully poured into ice-cold water (500 mL, 4 °C) to induce the precipitation of white flocculent solids. The crude product was collected through vacuum filtration using a Büchner funnel, followed by sequential washing with chilled water (3 × 100 mL) and cold diethyl ether (2 × 50 mL) to remove residual DMF and inorganic salts. Subsequent vacuum drying at 45 °C for 12 h afforded 8.90 g of white crystalline product, yielding 76.67%. M.p.:123.6–124.7 °C. ^1^H NMR (600 MHz, DMSO-*d*_6_) δ 9.78 (s, 1H), 7.74 (d, *J* = 1.5 Hz, 1H), 7.32 (d, *J* = 1.4 Hz, 1H), 6.27 (s, 2H). HRMS (ESI) *m*/*z*: 228.9484 ([M + H]^+^) (calcd for C_8_H_5_BrO_3_: 228.9501).

#### 2.1.3. Synthesis of Methyl 3-Bromo-4,5-methylenedioxybenzoate (**4**)

Iodine (I_2_, 11.53 g, 45.41 mmol) was dissolved in anhydrous methanol (100 mL), and the reaction system was immersed in an ice-salt bath (0 °C) to maintain thermal equilibrium. Compound **3** (8.00 g, 34.93 mmol) and potassium hydroxide (5.29 g, 94.31 mmol) were subsequently introduced into the mixture through a solid addition funnel in three equal portions at 10 min intervals, with the addition rate controlled to maintain the reaction temperature below 5 °C. The reaction proceeded under nitrogen atmosphere at 0 °C for 2 h. Completion of the reaction was confirmed by TLC analysis using a petroleum ether/ethyl acetate mixture (5:1, *v*/*v*) as the mobile phase. The reaction was quenched with ice-cold saturated ammonium chloride solution (NH_4_Cl, 50 mL) followed by removal of the cooling bath, and the mixture was stirred at 25 °C for 10 min. The solvent was removed under reduced pressure, and the residue was dispersed in ice water (50 mL). The resulting pale yellow precipitate was collected by vacuum filtration through a Büchner funnel, then sequentially washed with chilled water (3 × 30 mL) and cold methanol (2 × 10 mL) to eliminate residual iodides and inorganic salts. The purified solid was dried under vacuum at 40 °C for 8 h to afford 8.90 g of pale yellow crystalline product, corresponding to a yield of 96.2%. M.p.:96.0–97.6 °C. ^1^H NMR (600 MHz, DMSO-*d*_6_) δ 7.62 (d, *J* = 2.8 Hz, 1H), 7.34 (d, *J* = 2.9 Hz, 1H), 6.24 (s, 2H), 3.81 (s, 3H). HRMS (ESI) *m*/*z*: 258.9589 ([M + H]^+^) (calcd for C_9_H_7_BrO_4_: 258.9607).

#### 2.1.4. Synthesis of (*E*)-1-Bromo-3,5-dimethoxy-2-(2-nitrovinyl)benzene (**6**)

2-Bromo-4, 6-dimethoxybenzalhyde (compound **5**, 0.50 g, 2.04 mmol) was dissolved in anhydrous DCM (15 mL, HPLC-grade) under nitrogen atmosphere. The solution was treated with aqueous methylamine solution (CH_3_NH_2_·H_2_O, 0.10 g, 2.04 mmol) and stirred at room temperature (25°C) for 2.5 h. Subsequently, triethylamine (TEA, 0.20 g, 2.04 mmol) and nitromethane (CH_3_NO_2_, 0.20 g, 2.45 mmol) were added sequentially, followed by continuous stirring for 16 h. The reaction progress was monitored by TLC using a petroleum ether/ethyl acetate mixture (3:1, *v*/*v*) as the developing solvent until complete consumption of the starting materials was observed. After removal of the solvent under reduced pressure, the crude product was purified by column chromatography with petroleum ether/ethyl acetate (10:1, *v*/*v*) as the eluent to afford 4.01 g of the title compound as a white solid in 67.8% yield. M.p.: 112.4–114.5 °C. LC-MS *m*/*z*: 288.1 ([M + H]^+^).

#### 2.1.5. Synthesis of 3-(2-Bromo-4,6-dimethoxyphenyl)oxirane-2-carboxylic Acid (**9**)

A mixture of compound **5** (5.00 g, 16.32 mmol) and ethyl chloroacetate (ClCH_2_COOEt, 3.00 g, 24.48 mmol) in anhydrous tetrahydrofuran (THF, 40 mL) was cooled to 0 °C using an ice-salt bath. Sodium tert-butoxide (t-BuONa, 3.14 g, 32.64 mmol) and catalytic potassium iodide (0.05 g, 0.3 mmol, 2 mol%) were sequentially added in three portions at 5 min intervals through a solid addition funnel, maintaining strict temperature control (±2 °C). After complete addition, the reaction was stirred at 0 °C under nitrogen atmosphere for 2 h, with reaction progress monitored by TLC analysis (petroleum ether/ethyl acetate = 5:1, *v*/*v*). The cooled mixture was then treated dropwise with aqueous NaOH solution (1 M, 20 mL) via a pressure-equalizing dropping funnel, followed by continued stirring at 0 °C for 4 h. The resulting solution was carefully acidified to pH 6.5 using 1 M HCl and subsequently heated to 90 °C in a preheated oil bath for 3 h. Reaction completion was verified by TLC (eluent: DCM/methanol = 20:1, *v*/*v*). The cooled reaction mixture underwent extraction with ethyl acetate (3 × 50 mL), and the combined organic layers were washed with saturated brine (50 mL) and dried over anhydrous Na_2_SO_4_. After solvent removal under reduced pressure, the crude product was purified by column chromatography (DCM/methanol = 50:1, *v*/*v*) to afford 4.50 g of the title compound as a white solid in 66.6% yield. LC-MS *m*/*z*: 300.1 ([M − H]^−^).

#### 2.1.6. Synthesis of (*E*)-1-Bromo-3,5-dimethoxy-2-(2-methoxyvinyl)benzene (**10**)

Methoxymethyltriphenylphosphonium chloride (MMTPCl, 8.40 g, 12.24 mmol) was dissolved in anhydrous THF (20 mL) under nitrogen atmosphere. The reaction mixture was cooled to −20 °C and treated with a THF solution (15 mL) of potassium tert-butoxide (t-BuOK, 2.74 g, 12.24 mmol) via syringe pump at a controlled rate of 0.5 mL/min. After completion of the addition, the cooling bath was removed and the reaction was allowed to warm to ambient temperature (25 °C) with continuous stirring for 30 min to ensure complete formation of the ylide intermediate. The resulting ylide solution was recooled to −20 °C, followed by a slow infusion of a THF solution (10 mL) containing compound **5** (2.00 g, 8.16 mmol) through syringe pump at 0.2 mL/min. The reaction mixture was subsequently warmed to 25 °C and maintained under vigorous agitation for 6 h. Reaction progress was monitored by TLC using petroleum ether/ethyl acetate (3:1, *v*/*v*) as the mobile phase until complete consumption of the starting materials was observed. The reaction was quenched by careful addition of pre-cooled saturated ammonium chloride solution (30 mL, 4 °C). The organic phase was concentrated under reduced pressure, and the residue was triturated with ethyl acetate (3 × 50 mL). The combined organic extracts were transferred to a separatory funnel and washed sequentially with saturated brine solution (2 × 30 mL) to remove ionic byproducts. After drying over anhydrous sodium sulfate, the solution was concentrated in vacuo and purified by column chromatography using DCM/methanol (50:1, *v*/*v*) as eluent to afford the title compound as a white crystalline solid (1.89 g, 84.8% yield). M.p.: 125.3–127.5 °C. LC-MS *m*/*z*: 273.1 ([M + H]^+^).

#### 2.1.7. General Procedure for the Synthesis of 2-(4,4,5,5-Tetramethyl-1,3,2-dioxaborolan-2-yl)benzaldehyde Derivatives (**14a**–**14d**)

A mixture of 2-bromobenzaldehyde (compound **13a**, 5.00 g, 27.02 mmol) and bis(pinacolato) diboron (8.22 g, 32.43 mmol) was dissolved in a degassed 1,4-dioxane (100 mL). To this solution were sequentially added potassium acetate (KOAc, 7.96 g, 81.07 mmol) and dichloro [1,1′-bis(diphenylphosphino)ferrocene]palladium(II) (Pd(dppf)Cl_2_, 0.35 g, 0.48 mmol, 2.4 mol%). The reaction system was maintained at 80°C under nitrogen atmosphere for 12 h with continuous monitoring by TLC (eluent: petroleum ether/ethyl acetate 3:1, *v*/*v*) until complete consumption of the starting materials was observed. After cooling to ambient temperature (25 °C), the mixture was carefully quenched by pouring into ice-cold water (100 mL) and extracted with ethyl acetate (3 × 30 mL). The combined organic layers were washed successively with water (3 × 20 mL) and brine (30 mL) to remove residual inorganic salts, then dried over anhydrous sodium sulfate. The concentration under reduced pressure followed by column chromatography purification (silica gel; petroleum ether/ethyl acetate 5:1, *v*/*v*) afforded 2-(4,4,5,5-tetramethyl-1,3,2-dioxaborolan-2-yl)benzaldehyde (**14a**) as a yellow solid (3.80 g, 60,6% yield). ^1^H NMR (600 MHz, DMSO-*d*_6_) δ 10.35 (s, 1H), 7.91 (dd, *J* = 7.2, 1.9 Hz, 1H), 7.73 (dd, *J* = 7.0, 1.6 Hz, 1H), 7.70–7.63 (m, 2H), 1.34 (s, 12H). HRMS (ESI) *m*/*z*: 233.1332 ([M + H]^+^) (calcd for C_13_H_17_BO_3_: 233.1350).

4-methoxy-2-(4,4,5,5-tetramethyl-1,3,2-dioxaborolan-2-yl)benzaldehyde (**14b**) was prepared from Bis(pinacolato)diboron and 2-bromo-4-methoxybenzaldehyde, yielding 3.98 g of yellow solid with a yield of 65.8%. ^1^H NMR (600 MHz, DMSO-*d*_6_) δ 10.35 (s, 1H), 7.91 (dd, *J* = 7.2, 1.9 Hz, 1H), 7.73 (dd, *J* = 7.0, 1.6 Hz, 1H), 7.70–7.63 (m, 2H), 1.34 (s, 12H). HRMS (ESI) *m*/*z*: 263,1437 ([M + H]^+^) (calcd for C_14_H_19_BO_4_: 263.1455).

2,4-dimethoxy-6-(4,4,5,5-tetramethyl-1,3,2-dioxaborolan-2-yl)benzaldehyde (**14c**) was prepared from Bis(pinacolato)diboron and 2-bromo-4,6-dimethoxybenzaldehyde, yielding 4.01 g of yellow solid with a yield of 67.8%. ^1^H NMR (600 MHz, DMSO-*d*_6_) δ 10.11 (s, 1H), 6.68 (d, *J* = 1.5 Hz, 1H), 6.48 (d, *J* = 1.4 Hz, 1H), 3.91 (s, 3H), 3.87 (s, 3H), 1.31 (s, 12H). HRMS (ESI) *m*/*z*: 293,1541 ([M + H]^+^) (calcd for C_15_H_21_BO_5_: 293.1561).

4,5-dimethoxy-2-(4,4,5,5-tetramethyl-1,3,2-dioxaborolan-2-yl)benzaldehyde (**14d**) was prepared from Bis(pinacolato)diboron and 2-bromo-4,5-dimethoxybenzaldehyde, yielding 3.70 g of yellow solid with a yield of 62.1%. ^1^H NMR (600 MHz, DMSO-*d*_6_) δ 10.37 (s, 1H), 7.44 (s, 1H), 7.24 (s, 1H), 3.88 (s, 3H), 3.85 (s, 3H), 1.34 (s, 12H). HRMS (ESI) *m*/*z*: 293,1560 ([M + H]^+^) (calcd for C_15_H_21_BO_5_: 293.1561).

#### 2.1.8. General Procedure for the Synthesis of Methyl 7-(2-Formylphenyl)benzo[*d*][1,3]dioxole-5-carboxylate Derivatives (**15a**–**15d**)

A mixture of methyl 3-bromo-4,5-methylenedioxybenzoate (compound **4**, 4.00 g, 15.44 mmol) and 2-(4,4,5,5-tetramethyl-1,3,2-dioxaborolan-2-yl)benzaldehyde (compound **14a**, 3.94 g, 16.98 mmol) was dissolved in a degassed 1,4-dioxane/water solution (40 mL/10 mL, *v*/*v* 4:1). To this solution were sequentially added cesium carbonate (Cs_2_CO_3_, 15.09 g, 46.32 mmol) and Tetrakis(triphenylphosphine)palladium(0) (Pd(PPh_3_)_4_, 0.35 g, 0.48 mmol, 2.4 mol%). The reaction system was maintained at 80°C under nitrogen atmosphere for 12 h with continuous monitoring by TLC (eluent: petroleum ether/ethyl acetate 3:1, *v*/*v*) until complete consumption of the starting materials was observed. After cooling to ambient temperature (25 °C), the mixture was carefully quenched by pouring into ice-cold water (100 mL) and extracted with ethyl acetate (3 × 30 mL). The combined organic layers were washed successively with water (3 × 20 mL) and brine (30 mL) to remove residual inorganic salts, then dried over anhydrous sodium sulfate. The concentration under reduced pressure followed by column chromatography purification (silica gel; petroleum ether/ethyl acetate 5:1, *v*/*v*) afforded methyl 7-(2-formylphenyl)benzo[*d*][1,3]dioxole-5-carboxylate (**15a**) as a yellow solid (3.58 g, 81.6% yield). ^1^H NMR (600 MHz, DMSO-*d*_6_) δ 9.92 (s, 1H), 9.93–9.88 (m, 1H), 7.92–7.98 (m, 1H), 7.76–7.82 (m, 1H), 7.68–7.62 (m, 1H), 7.58 (d, *J* = 1.5 Hz, 1H), 7.48 (d, *J* = 1.5 Hz, 1H), 6.16 (s, 2H), 3.84 (s, 3H). HRMS (ESI) *m*/*z*: 285.0743 ([M + H]^+^) (calcd for C_16_H_12_O_5_: 285.0764).

Methyl 7-(2-formyl-5-methoxyphenyl)benzo[*d*][1,3]dioxole-5-carboxylate (**15b**) was prepared from methyl 3-bromo-4,5-methylenedioxybenzoate and 4-methoxy-2-(4,4,5,5-tetramethyl-1,3,2-dioxaborolan-2-yl)benzaldehyde, yielding 3.87 g of yellow solid with a yield of 79.8%. ^1^H NMR (600 MHz, DMSO-*d*_6_) δ 9.75 (s, 1H), 7.92 (d, *J* = 8.7 Hz, 1H), 7.58 (d, *J* = 1.7 Hz, 1H), 7.48 (d, *J* = 1.9 Hz, 1H), 7.19 (dd, *J* = 8.8, 2.6 Hz, 1H), 7.03 (d, *J* = 2.6 Hz, 1H), 6.15 (s, 2H), 3.90 (s, 3H), 3.83 (s, 3H). HRMS (ESI) *m*/*z*: 315.0848 ([M + H]^+^) (calcd for C_17_H_14_O_6_: 315.0869).

Methyl 7-(2-formyl-3,5-dimethoxyphenyl)benzo[*d*][1,3]dioxole-5-carboxylate (**15c**) was prepared from methyl 3-bromo-4,5-methylenedioxybenzoate and 2,4-dimethoxy-6-(4,4,5,5-tetramethyl-1,3,2-dioxaborolan-2-yl)benzaldehyde, yielding 4.30 g of yellow solid with a yield of 80.9%. ^1^H NMR (600 MHz, DMSO-*d*_6_) δ 10.14 (s, 1H), 7.44 (d, *J* = 1.6 Hz, 1H), 7.39 (d, *J* = 1.6 Hz, 1H), 6.78 (d, *J* = 2.3 Hz, 1H), 6.51 (d, *J* = 2.3 Hz, 1H), 6.06 (s, 2H), 3.93 (s, 3H), 3.89 (s, 3H), 3.81 (s, 3H). HRMS (ESI) *m*/*z*: 345.0952 ([M + H]^+^) (calcd for C_18_H_16_O_7_: 345.0975).

Methyl 7-(2-formyl-4,5-dimethoxyphenyl)benzo[*d*][1,3]dioxole-5-carboxylate (**15d**) was prepared from methyl 3-bromo-4,5-methylenedioxybenzoate and 4,5-dimethoxy-2-(4,4,5,5-tetramethyl-1,3,2-dioxaborolan-2-yl)benzaldehyde, yielding 4.32 g of yellow solid with a yield of 81.3%. ^1^H NMR (600 MHz, DMSO-*d*_6_) δ 9.73 (s, 1H), 7.59 (d, *J* = 1.7 Hz, 1H), 7.46 (d, *J* = 1.6 Hz, 1H), 7.42 (s, 1H), 7.05 (s, 1H), 6.14 (s, 2H), 3.90 (s, 3H), 3.87 (s, 3H), 3.83 (s, 3H). HRMS (ESI) *m*/*z*: 345.0969 ([M + H]^+^) (calcd for C_18_H_16_O_7_: 345.0975).

#### 2.1.9. General Procedure for the Synthesis of Methyl (*E*)-7-[2-(2-Methoxyvinyl)phenyl]benzo[*d*][1,3]dioxole-5-carboxylate Derivatives (**16a**–**16d**)

A solution of MMTPCl (7.96 g, 23.22 mmol) in anhydrous THF (20 mL) was charged into a pre-dried 250 mL three-necked flask equipped with a constant-pressure dropping funnel. The reaction system was cooled to −20 °C under nitrogen atmosphere. A THF solution (15 mL) of potassium tert-butoxide (t-BuOK, 2.61 g, 23.22 mmol) was subsequently introduced via the dropping funnel at a controlled rate of 0.5 mL/min. Following complete addition, the cooling bath was removed to allow gradual warming to ambient temperature (25 °C) with continuous stirring maintained for 30 min to ensure complete generation of the ylide intermediate. The resulting intermediate was then treated with a solution of methyl 7-(2-formylphenyl)benzo[*d*][1,3]dioxole-5-carboxylate (compound **15a**, 3.00 g, 10.55 mmol) in anhydrous THF (10 mL), delivered through a syringe pump at 0.2 mL/min. The reaction mixture was vigorously stirred at 25 °C for 6 h, with progress monitored by TLC (eluent: petroleum ether/ethyl acetate = 3:1, *v*/*v*) until complete consumption of starting materials was observed. The reaction was quenched by the cautious addition of ice-cold saturated ammonium chloride solution (30 mL, 4 °C), followed by solvent removal under reduced pressure. The residue was sequentially dispersed in ethyl acetate (3 × 50 mL) and transferred to a separatory funnel. The organic layer was washed with saturated brine solution (2 × 30 mL) to remove inorganic salts, dried over anhydrous sodium sulfate, and concentrated under vacuum to afford methyl (*E*)-7-[2-(2-methoxyvinyl)phenyl]benzo[*d*][1,3]dioxole-5-carboxylate (**16a**) as a yellow solid (2.19 g, 66.4% yield). LC-MS *m*/*z*: 313.1 ([M + H]^+^). The crude product was used directly in the next step without further purification.

Methyl (*E*)-7-[5-methoxy-2-(2-methoxyvinyl)phenyl]benzo[*d*][1,3]dioxole-5-carboxylate (**16b**) was prepared from methyl 7-(2-formyl-5-methoxyphenyl)benzo[*d*][1,3]dioxole-5-carboxylate, yielding 2.27 g of yellow solid with a yield of 69.7%. LC-MS *m*/*z*: 343.3 ([M + H]^+^).

Methyl (*E*)-7-[3,5-dimethoxy-2-(2-methoxyvinyl)phenyl]benzo[*d*][1,3]dioxole-5-carboxylate (**16c**) was prepared from methyl 7-(2-formyl-3,5-dimethoxyphenyl)benzo[*d*][1,3]dioxole-5-carboxylate, yielding 2.21 g of yellow solid with a yield of 68.1%. LC-MS *m*/*z*: 373.4 ([M + H]^+^).

Methyl (*E*)-7-[2-(2-methoxyvinyl)-4,5-dimethoxyphenyl]benzo[*d*][1,3]dioxole-5-carboxylate (**16d**) was prepared from methyl 7-(2-formyl-4,5-dimethoxyphenyl)benzo[*d*][1,3]dioxole-5-carboxylate, yielding 2.18 g of yellow solid with a yield of 67.2%. LC-MS *m*/*z*: 373.5 ([M + H]^+^).

#### 2.1.10. General Procedure for the Synthesis of Methyl Phenanthro [3,4-d][1,3]dioxole-5-Carboxylate Derivatives (**17a**–**17d**)

A mixture of methyl (*E*)-7-[2-(2-methoxyvinyl)phenyl]benzo[*d*][1,3]dioxole-5-carboxylate (compound **16a**, 1.50 g, 4.03 mmol) and bismuth triflate (Bi(OTf)_3_, 0.16 g, 0.24 mmol, 6.0 mol%) was charged into a pre-dried 100 mL three-necked flask containing anhydrous 1,2-dimethoxyethane (DME, 20 mL). Under nitrogen atmosphere, the mixture was heated to 30 °C and maintained at this temperature with continuous stirring for 2 h. The reaction progress was periodically analyzed by TLC (eluent: petroleum ether/ethyl acetate = 4:1, *v*/*v*) until complete disappearance of the starting materials was confirmed. Following confirmation of reaction completion, the solvent was removed under reduced pressure. The crude product was purified through column chromatography using DCM as eluent, yielding methyl phenanthro [3,4-d][1,3]dioxole-5-carboxylate as a pale yellow solid (1.19 g, 88.4% yield). ^1^H NMR (600 MHz, DMSO-*d*_6_) δ 9.07–9.01 (m, 1H), 8.81 (d, *J* = 9.5, 1H), 8.77–8.83 (m, 1H), 7.94–7.98 (m, 1H), 7.89 (s, 1H), 7.80 (d, *J* = 9.5, 1H), 7.65–7.69 (m, 2H), 6.45 (s, 2H), 3.92 (s, 3H). HRMS (ESI) *m*/*z*: 281.0795 ([M + H]^+^) (calcd for C_17_H_12_O_4_: 281.0815).

Methyl 10-methoxyphenanthro [3,4-d][1,3]dioxole-5-carboxylate (**17b**) was prepared from methyl (*E*)-7-[5-methoxy-2-(2-methoxyvinyl)phenyl]benzo[*d*][1,3]dioxole-5-carboxylate, yielding 1.23 g of yellow solid with a yield of 90.5%. ^1^H NMR (600 MHz, DMSO-*d*_6_) δ 8.54–8.47 (m, 1H), 7.92 (d, *J* = 8.7 Hz, 1H), 7.85 (s, 1H), 7.74 (d, *J* = 9.4 Hz, 1H), 7.36 (dd, *J* = 8.8, 2.5 Hz, 1H), 6.44 (s, 2H), 3.93 (s, 3H), 3.92 (s, 3H). HRMS (ESI) *m*/*z*: 311.0920 ([M + H]^+^) (calcd for C_18_H_14_O_5_: 311.0920).

Methyl 8,10-dimethoxyphenanthro [3,4-d][1,3]dioxole-5-carboxylate (**17c**) was prepared from methyl (*E*)-7-[3,5-dimethoxy-2-(2-methoxyvinyl)phenyl]benzo[*d*][1,3]dioxole-5-carboxylate, yielding 1.13 g of yellow solid with a yield of 82.4%. ^1^H NMR (600 MHz, DMSO-*d*_6_) δ 8.49 (d, *J* = 9.6 Hz, 1H), 8.16 (d, *J* = 2.0 Hz, 1H), 7.98 (d, *J* = 9.4 Hz, 1H), 7.88 (s, 1H), 6.90 (d, *J* = 2.2 Hz, 1H), 6.44 (s, 2H), 4.00 (s, 3H), 3.93 (s, 3H), 3.91 (s, 3H). HRMS (ESI) *m*/*z*: 341.1004 ([M + H]^+^) (calcd for C_19_H_16_O_6_: 341.1026).

Methyl 9,10-dimethoxyphenanthro [3,4-d][1,3]dioxole-5-carboxylate (**17d**) was prepared from methyl (*E*)-7-[4,5-dimethoxy-2-(2-methoxyvinyl)phenyl]benzo[*d*][1,3]dioxole-5-carboxylate, yielding 1.22 g of yellow solid with a yield of 88.9%. ^1^H NMR (600 MHz, DMSO-*d*_6_) δ 8.55 (d, *J* = 9.4 Hz, 1H), 8.53 (s, 1H), 7.83 (s, 1H), 7.75 (d, *J* = 9.3 Hz, 1H), 7.50 (s, 1H), 6.44 (s, 2H), 3.94 (s, 6H), 3.92 (s, 3H). HRMS (ESI) *m*/*z*: 341.1018 ([M + H]^+^) (calcd for C_19_H_16_O_6_: 341.1026).

#### 2.1.11. General Procedure for the Synthesis of Phenanthro [3,4-d][1,3]dioxole-5-Carboxylic Acid Derivatives (**18a**–**18d**)

A mixture of methyl phenanthro [3,4-d][1,3]dioxole-5-carboxylate (compound **17a**, 1.00 g, 3.57 mmol) and sodium hydroxide (0.29 g, 7.14 mmol) was charged into a 100 mL three-necked flask, followed by the addition of water (20 mL). The reaction system was heated to 30 °C under atmospheric conditions and maintained at this temperature with continuous stirring for 12 h. Complete consumption of the starting materials was confirmed by TLC (eluent: DCM/methanol = 10:1, *v*/*v*). The reaction mixture was subsequently cooled in an ice bath, and concentrated hydrochloric acid (HCl, 37% *w*/*w*) was carefully introduced via a constant-pressure dropping funnel to adjust the pH to 2.0, resulting in the formation of white flocculent precipitate. After continued stirring for 30 min, the solid product was collected by vacuum filtration using a Büchner funnel. The filter cake was washed with ice-cold water (3 × 10 mL) to remove residual inorganic salts, followed by drying in a vacuum oven at 40 °C for 8 h to afford phenanthro [3,4-d][1,3]dioxole-5-carboxylic acid as white crystalline solid (0.79 g, 83.2% yield). ^1^H NMR (600 MHz, DMSO-*d*_6_) δ 13.20 (s, 1H), 9.07–9.01 (m, 1H), 8.81 (d, *J* = 9.5, 1H), 8.77–8.83 (m, 1H), 7.94–7.98 (m, 1H), 7.89 (s, 1H), 7.80 (d, *J* = 9.5, 1H), 7.65–7.69 (m, 2H), 6.45 (s, 2H); ^13^C NMR (151 MHz, DMSO-*d*_6_) δ 168.66, 146.98, 144.93, 131.73, 128.38, 128.26, 127.97, 127.85, 127.42, 127.33, 127.06, 124.42, 122.61, 116.12, 112.40, 102.82. HRMS (ESI) *m*/*z*: 265.0513 ([M − H]^−^), (calcd for C_16_H_10_O_4_: 265.0500).

10-Methoxyphenanthro [3,4-d][1,3]dioxole-5-carboxylic acid (**18b**) was prepared from methyl 10-methoxyphenanthro [3,4-d][1,3]dioxole-5-carboxylate, yielding 0.83 g of white solid with a yield of 86.9%. ^1^H NMR (600 MHz, DMSO-*d*_6_) δ 13.09 (s, 1H), 8.66 (d, *J* = 9.4 Hz, 1H), 8.54 (d, *J* = 2.5 Hz, 1H), 7.92 (d, *J* = 8.8 Hz, 1H), 7.88 (s, 1H), 7.73 (d, *J* = 9.4 Hz, 1H), 7.36 (dd, *J* = 8.7, 2.6 Hz, 1H), 6.44 (s, 2H), 3.93 (s, 3H); ^13^C NMR (151 MHz, DMSO-*d*_6_) δ 168.69, 158.51, 147.06, 144.48, 129.78, 129.17, 128.72, 127.06, 126.20, 122.32, 121.93, 117.17, 115.88, 112.54, 109.28, 102.78, 55.78. HRMS (ESI) *m*/*z*: 295.0611 ([M − H]^−^) (calcd for C_17_H_12_O_5_: 295.0606).

8,10-Dimethoxyphenanthro [3,4-d][1,3]dioxole-5-carboxylic acid (**18c**) was prepared from methyl 8,10-dimethoxyphenanthro [3,4-d][1,3]dioxole-5-carboxylate, yielding 0.82 g of white solid with a yield of 85.5%. ^1^H NMR (600 MHz, DMSO-*d*_6_) δ 13.14 (s, 1H), 8.63 (d, *J* = 9.7 Hz, 1H), 8.15 (d, *J* = 2.5 Hz, 1H), 7.95 (d, *J* = 9.6 Hz, 1H), 7.87 (s, 1H), 6.88 (d, *J* = 2.5 Hz, 1H), 6.42 (s, 2H), 3.99 (s, 3H), 3.93 (s, 3H); ^13^C NMR (151 MHz, DMSO-*d*_6_) δ 168.67, 159.31, 156.38, 147.15, 144.37, 129.76, 129.11, 122.25, 121.14, 120.41, 117.27, 116.01, 112.83, 102.67, 100.86, 98.73, 56.38, 55.80. HRMS (ESI) *m*/*z*: 325.0710 ([M − H]^−^), (calcd for C_18_H_14_O_6_: 325.0711).

9,10-Dimethoxyphenanthro [3,4-d][1,3]dioxole-5-carboxylic acid (**18d**) was prepared from methyl 9,10-dimethoxyphenanthro [3,4-d][1,3]dioxole-5-carboxylate, yielding 0.78 g of white solid with a yield of 81.4%. ^1^H NMR (600 MHz, DMSO-*d*_6_) δ 13.08 (s, 1H), 8.72–8.67 (m, 1H), 8.49 (d, *J* = 18.5 Hz, 1H), 7.85–7.78 (m, 1H), 7.72 (d, *J* = 16.3 Hz, 1H), 7.52–7.42 (m, 1H), 6.45–6.36 (m, 2H), 3.90 (s, 6H); ^13^C NMR (151 MHz, DMSO-*d*_6_) δ 168.76, 149.78, 149.18, 146.14, 144.19, 127.74, 127.15, 126.74, 122.45, 122.33, 122.13, 116.12, 111.81, 108.76, 108.15, 102.59, 55.96, 55.94. HRMS (ESI) *m*/*z*: 325.0726 ([M − H]^−^) (calcd for C_18_H_14_O_6_: 325.0711).

### 2.2. Cytotoxicity of Denitroaristolochic Acids in HK2 Cells

This study systematically investigated the cytotoxic effects and structure-activity relationships of denitroaristolochic acid analogs (DAA-II, DAA-III, DAA-IV, DAA-V) on human renal cortical proximal tubular epithelial cells (HK2) using the cell counting kit-8 (CCK-8) assay. HK2 cells were maintained in Dulbecco’s modified eagle medium (DMEM) supplemented with 10% fetal bovine serum under standard culture conditions (37 °C, 5% CO_2_). At 80% confluence, cells were trypsinized and seeded into 96-well plates at a density of 1 × 10^4^ cells/well, followed by a 4–6 h attachment period prior to compound exposure. Stock solutions (50 mM) of each DAA were prepared in DMSO and serially diluted to final concentrations ranging from 3 to 1000 μM, with DMSO concentration strictly maintained at 0.5% (*v*/*v*) in all treatment groups. Experimental controls included AA-I as positive control and 0.5% DMSO-containing medium as vehicle control. Following 24 h compound exposure, cell viability was assessed using CCK-8 reagent according to manufacturer specifications: 10 μL CCK-8 solution was added to each well, followed by 2 h incubation under light-protected conditions. Absorbance was measured at 450 nm using a microplate reader, with morphological observations conducted via inverted phase-contrast microscopy.

## 3. Results

### 3.1. Synthesis of Four Denitroaristolochic Acids

**Figure 1:**
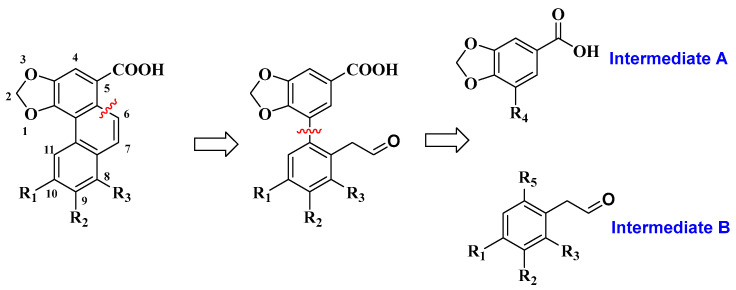
Retrosynthetic analysis of denitroaristolochic acids.

Up to now, there have been no reports on the synthesis of target denitroaristolochic acids. This study developed an efficient synthesis of denitroaristolochic acids via retrosynthetic disconnection at C-4/C-10, disconnecting the target compounds into aryl bromide (intermediate A) and boronic ester (intermediate B). Intermediate A was prepared through regioselective Br_2_-mediated electrophilic bromination under protic acid catalysis. Intermediate B was prepared via Pd(dppf)_2_Cl_2_-catalyzed Miyaura borylation of aryl iodide with bis(pinacolato)diboron (B_2_pin_2_) in anhydrous DME. The biaryl framework was directionally constructed by Suzuki–Miyaura coupling of A and B using Pd(dppf)_2_Cl_2_/Cs_2_CO_3_ in 1,4-dioxane/water (4:1) at 100 °C for 16 h. This modular strategy, integrating electronic modulation and coupling–cyclization sequences, offers a scalable approach for functional natural product derivatization (Figure 1).

**Scheme 1:**
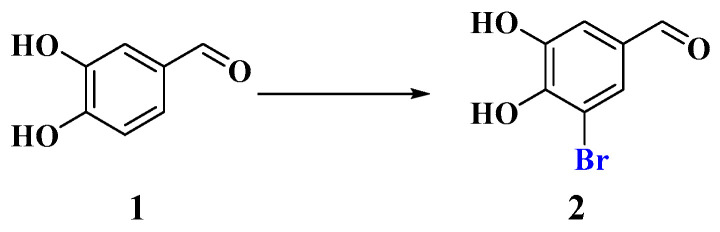
Synthesis of compound **2**.

Based on previous investigations demonstrating the regioselective bromination pattern of piperonal (preferential substitution at aldehyde-adjacent positions), 3,4-dihydroxybenzoic acid was strategically selected as the starting material to circumvent potential side reactions associated with aldehyde-directed ortho substitution. The catechol moiety (3,4-dihydroxy groups) in this substrate significantly enhances the nucleophilic susceptibility at the meta-position (C-5) through pronounced electron-donating effects. The bromination procedure was carried out by dissolving 3,4-dihydroxybenzaldehyde in acetic acid, followed by a gradual addition of bromine (Br_2_) at ambient temperature (25 °C). After 6 h of reaction under electrophilic bromination conditions, selective incorporation of a bromine atom at the C-5 position successfully afforded 5-bromo-3,4-dihydroxybenzoic acid (compound **2**) with controlled regioselectivity (Scheme 1).

Comparative analysis of reaction conditions in Table 2 revealed that Entry 9 demonstrated superior performance, achieving a 50.8% yield under catalyst-free conditions using bromine (1.1 equiv) in glacial acetic acid at room temperature for 6 h. In contrast, other experimental groups employing either *N*-bromosuccinimide (NBS) or dibromohydantoin as brominating agents with *m*-chloroperbenzoic acid (*m*-CPBA) catalyst in methanol, DCM, or THF (Entries 1–6), along with those omitting catalysts but extending reaction duration to 12 h (Entries 7–8), failed to achieve complete conversion or satisfactory yields. This comparative data suggests that elemental bromine exhibits higher electrophilic reactivity, while the high-polarity acetic acid solvent effectively facilitates the reaction process. Notably, the introduced catalyst appears to have exerted inhibitory effects on the primary reaction pathway. Furthermore, the reduced reaction time of 6 h potentially minimizes competing side reactions, thereby enhancing process efficiency.

The optimized protocol therefore comprises bromine as the bromination reagent, glacial acetic acid as the reaction medium, catalyst-free system, and a 6 h reaction duration at ambient temperature. This streamlined system demonstrates significant yield improvement through rational reagent–solvent compatibility and process simplification, establishing a strategic foundation for subsequent synthetic optimization.

**Scheme 2:**
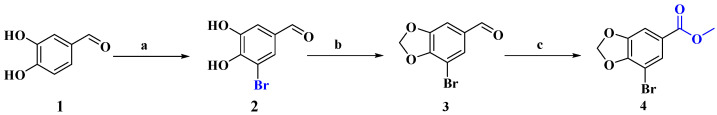
Synthesis of compound **4**. Reagents and conditions: (a) Br_2_, ethyl acetate, r.t., 6h, 70.1%; (b) CH_2_Br_2_, K_2_CO_3_, DMF, 110 °C, 4 h, 76.7%; (c) I_2_, KOH, CH_3_OH, 0 °C, 2 h, 96.2%.

The observed regioselectivity arises from synergistic effects involving p-π conjugation in catechol hydroxyl groups and protonation stabilization by acetic acid solvent. Specifically, the p-π conjugation enhances electron density at the aldehyde group’s meta-position, while the solvent-mediated protonation stabilizes the brominated transition state. Subsequent construction of the oxygen heterocyclic framework was accomplished via nucleophilic cyclization between the catechol oxyanion and dibromomethane (CH_2_Br_2_). For the synthesis of compound **3**, a mixture of compound **2** and CH_2_Br_2_ (1.2 equiv) was stirred in anhydrous DMF with K_2_CO_3_ (3.0 equiv) at 110 °C under nitrogen atmosphere for 5 h. Under these alkaline conditions, the 3,4-dihydroxy groups underwent bimolecular nucleophilic substitution (S_N_2) with CH_2_Br_2_, generating bicarbonate and bromide ions while forming the methylene-bridged benzodioxole structure. The final iodination–oxidation sequence to synthesize compound **4** was performed under strict temperature control. Molecular iodine (1.5 equiv) was dissolved in methanol, followed by sequential addition of KOH (2.0 equiv) and compound **3** under ice-bath conditions (0 °C). The reaction mixture was vigorously stirred at 0–5 °C for 2 h, achieving consecutive iodination and oxidation to yield compound **4** (intermediate A) (Scheme 2).

The key nitroolefin structure was constructed through a Henry (nitroaldol) reaction sequence. Starting with compound **5**, the synthetic protocol commenced with condensation of the aldehyde group with aqueous methylamine (33% *w*/*w*) in DCM at ambient temperature (25 °C) for 2.5 h, generating the corresponding imine intermediate. Subsequently, triethylamine and nitromethane were introduced to facilitate a 16 h cascade reaction involving nitroaldol addition followed by β-elimination, stereoselectively yielding (*E*)-1-bromo-3,5-dimethoxy-2-(2-nitrovinyl)benzene (compound **6**). Mechanistically, triethylamine served dual functions: neutralization of acidic byproducts from the condensation step to drive reaction completion, while simultaneously creating a basic environment that promoted the conjugate addition–elimination sequence. The pronounced electron-withdrawing nature of the nitro group stabilized the (*E*)-configured transition state through conjugation effects, thereby thermodynamically favoring the formation of the trans-nitroolefin product.

**Scheme 3:**
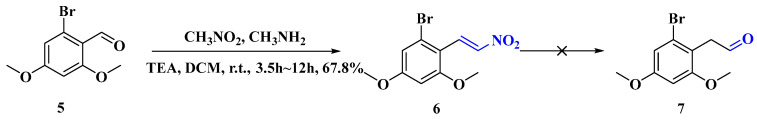
Synthesis of compound **7** via nitromethane nucleophilic substitution followed by amination.

Systematic evaluation of conventional reduction systems for transforming compound **6** to intermediate B revealed inherent limitations in achieving concurrent nitro group reduction and olefin preservation. Zinc dust in hydrochloric acid (Zn/HCl, 0 °C → reflux, 6 h) effected partial reduction to the hydroxylamine stage (–NHOH) without vinyl hydrogenation. While iron powder in acetic acid (Fe/AcOH, 80 °C, 12 h) accomplished complete nitro-to-amine conversion (–NH_2_), this protocol suffered from unsatisfactory yields (<10%). Tin(II) chloride in hydrochloric acid (SnCl_2_/HCl, ethanol reflux, 8 h) induced over-reduction in the nitro group alongside non-selective hydrogenation of the vinyl moiety to a saturated methylene unit. These empirical observations underscore the intrinsic challenges of conventional reductants in balancing nitro reduction depth with olefin hydrogenation selectivity, likely attributable to indiscriminate reactivity under strongly acidic conditions or excessive reduction potentials that compromise sensitive functional groups (Scheme 3).

Given the aforementioned challenges, the synthetic strategy was revised to construct the epoxide–carboxylate intermediate via Darzens condensation followed by decarboxylation to obtain intermediate B. The experimental procedure was performed as follows: a mixture of compound **5** and ethyl chloroacetate in THF was treated with sodium tert-butoxide (t-BuONa) as a strong base at strictly controlled 0 °C. The reaction proceeded through a nucleophilic addition–elimination mechanism, where t-BuONa selectively deprotonated the α-carbon of ethyl chloroacetate to generate a stabilized carbanion intermediate. This nucleophilic species underwent stereoselective addition to the aldehyde group, forming a β-hydroxy-ethyl carboxylate oxyanion intermediate. Subsequent intramolecular nucleophilic substitution (S_N_2) at the β-chloride position induced C–Cl bond heterolysis with concomitant stereochemical inversion, ultimately constructing the epoxide ring to yield ethyl 3-(2-bromo-4,6-dimethoxyphenyl)oxirane-2-carboxylate (compound **8**).

**Scheme 4:**
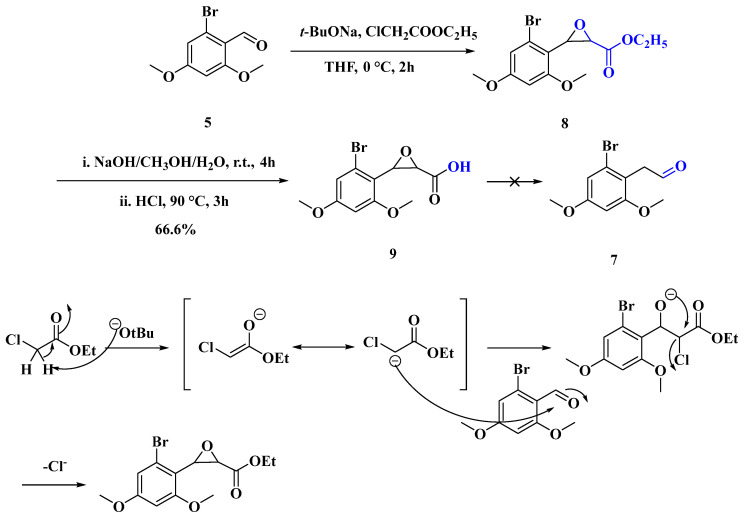
Synthesis of compound **7** via the Darzens reaction. Reaction mechanism from compound **5** to compound **8**.

The obtained compound **8** was then subjected to saponification in methanol/water (3:1, *v*/*v*) with sodium hydroxide at room temperature for 4 h, producing the corresponding sodium carboxylate intermediate. Subsequent acidification with hydrochloric acid to pH 2–3 followed by reflux at 90 °C for 3 h afforded compound **9**. However, neither the expected intermediate B (compound **7**) nor the side product resulting from ring-opening at the alternative position of the epoxide ring was observed during the decarboxylation process of compound **9**, indicating incomplete conversion under the current reaction conditions (Scheme 4).

According to comparative experimental data presented in Table 3, reactions employing sulfuric, hydrochloric, or phosphoric acid in THF/DCM mixed solvents under reflux conditions (Entry 1–9) consistently demonstrated yields below 10%. This observation suggests that the current combination of acidic reagents and solvent systems may fail to effectively promote the target reaction, potentially due to excessive acidity promoting side reactions or inadequate solvation capacity/stability of the solvent system for the reactants. Notably, Entry 10 employing diphenyl ether as a solvent at elevated temperature (260 °C) resulted in new impurity formation.

Critical analysis reveals three fundamental limitations in the current system: (1) inappropriate selection of acidic reagents, (2) insufficient thermal stability of solvent systems, and (3) suboptimal compatibility between reaction components and conditions. Subsequent optimization should prioritize the following: (i) investigation of weak acid or buffered systems to modulate reaction acidity; (ii) evaluation of thermally stable solvents such as DMSO or ionic liquids; and (iii) exploration of mild catalytic systems.

Furthermore, implementing pH regulation within a moderate range (pH 3–5) and establishing staged reaction control strategies (e.g., temperature gradients) may enhance reaction efficiency and selectivity. Systematic optimization focusing on the synergistic effects of reagent selection, solvent properties, and temperature–time parameters could potentially overcome the current yield limitations. Particular attention should be paid to balancing acid strength with substrate stability while maintaining effective mass transfer throughout the reaction process.

The synthetic route of intermediate B was redesigned employing a Wittig reaction to construct the methoxyvinyl aromatic framework. Under nitrogen atmosphere, a solution of MMTPCl in anhydrous THF was gradually added to potassium tert-butoxide (t-BuOK) dissolved in anhydrous THF at ambient temperature (25 °C). After complete addition, the mixture was vigorously stirred for 30 min to generate the phosphorus ylide intermediate. The reaction system was subsequently cooled to 0 °C, followed by dropwise addition of compound **5** dissolved in THF. The resulting mixture was then warmed to room temperature and maintained for 6 h, during which the (*E*)-1-bromo-3,5-dimethoxy-2-(2-methoxyvinyl)benzene (compound **10**) was stereoselectively formed through a nucleophilic addition–elimination mechanism between the ylide and aldehyde group.

**Scheme 5:**
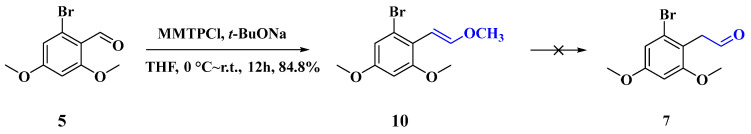
Synthesis of compound **7** via the Wittig reaction.

For the subsequent transformation to intermediate B, acid-mediated hydrolysis conditions were systematically investigated. Compound **10** was dissolved in THF and treated with 5 N HCl (3.0 equiv) at 65 °C for 8 h. LC-MS analysis revealed that the starting material remained predominantly unreacted (>95% recovery) with trace amounts of a demethoxylated byproduct (observed [M + H]^+^ at *m*/*z* 289.02). This observation indicates remarkable chemical inertness of the methoxyvinyl moiety under strongly acidic conditions, which could be rationalized through three fundamental aspects: (1) Electronic stabilization effect from the electron-donating methoxy groups reduces the electrophilicity of the conjugated double bond. (2) Steric hindrance arising from the adjacent bromine atom and two methoxy substituents impedes protonation of the vinyl group. (3) Thermodynamic stabilization of the extended conjugation system (aromatic ring-vinyl-methoxy) diminishes the driving force for hydrolysis (Scheme 5).

**Scheme 6:**
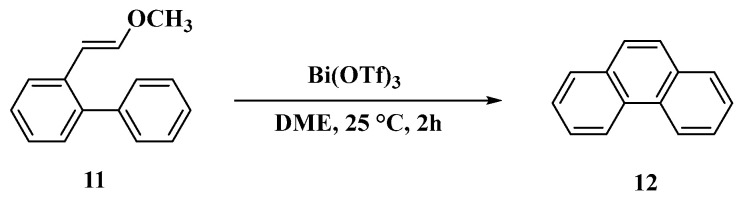
Construction of the phenanthrene ring via bismuth trifluoroacetate.

Masahito Murai et al. [30] proposed an efficient synthetic strategy for constructing polycyclic aromatic hydrocarbons based on Lewis acid-catalyzed regioselective cyclization. In this methodology, 2-(2-arylphenyl)vinyl ether precursors underwent intramolecular Friedel–Crafts cyclization under mild conditions (room temperature, solvent-free, or low-polarity solvent systems) mediated by bismuth triflate (Bi(OTf)_3_, 0.05 equiv). Experimental results demonstrated that the oxygen atom of the vinyl ether moiety synergistically directed the regioselective formation of substituted phenanthrene frameworks through an electrophilic aromatic activation mechanism, achieving high yields ranging from 82% to 95%. Mechanistic investigations revealed that the observed regioselectivity originated from the specific coordination activation of the vinyl ether oxygen atom by Bi(OTf)_3_, which preferentially promoted electrophilic substitution at the ortho-position of the aromatic ring while effectively suppressing competing para- and meta-cyclization pathways (Scheme 6).

An efficient synthetic strategy was successfully developed for the preparation of denitroaristolochic acid II, III, IV, and V. The synthesis commenced with a palladium-catalyzed Suzuki–Miyaura cross-coupling reaction between brominated compounds **13a**–**13d** and bis(pinacolato)diboron, affording the arylboronic ester derivatives **14a**–**14d**. This reaction required strictly anhydrous and oxygen-free conditions, proceeding in dioxane at 80 °C for 12 h. Subsequently, a palladium-catalyzed Suzuki–Miyaura cross-coupling reaction between aryl bromide **4** and arylboronic esters **14a**–**14d** was performed. This transformation was conducted in a degassed 4:1 (*v*/*v*) dioxane/water mixture for 12 h at 80 °C under a nitrogen atmosphere. Employing tetrakis(triphenylphosphine)palladium(0) (Pd(PPh_3_)_4_, 5 mol%) as the catalyst and Cs_2_CO_3_ (3.0 equiv) as the base, the reaction proceeded via the well-established catalytic cycle involving oxidative addition, transmetalation, and reductive elimination, yielding the biaryl compounds **15a**–**15d**.

Subsequently, the Wittig olefination was performed by first generating the active phosphorus ylide through treatment of MMTPCl (1.2 equiv) with potassium tert-butoxide (1.5 equiv) in anhydrous THF at 25 °C under nitrogen atmosphere. After 1 h activation, compounds **15a**–**15d** (1.0 equiv) in THF were added dropwise to the pre-cooled (0 °C) ylide solution, followed by gradual warming to ambient temperature with continued stirring for 6 h to furnish (*E*)-methoxyvinyl derivatives **16a**–**16d**.

**Scheme 7:**
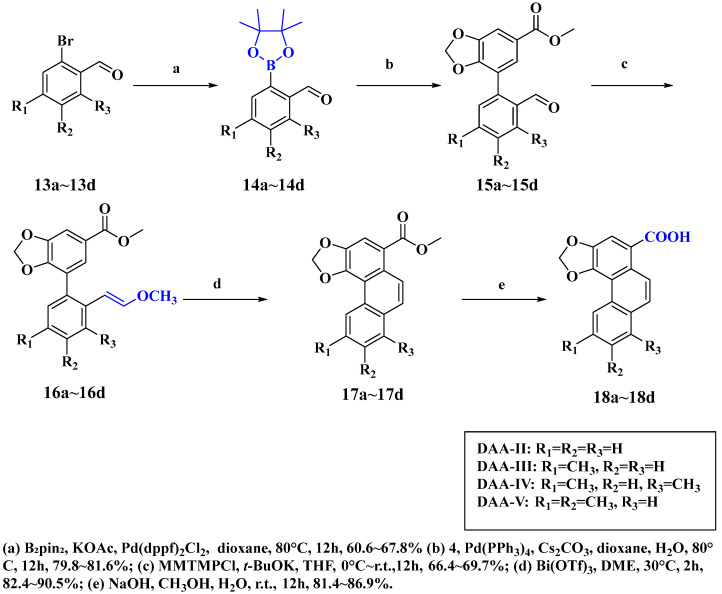
Synthesis of compounds **18a**–**18d** from compounds **13a**–**13d**.

The critical annulation step was achieved through bismuth(III)-mediated intramolecular Friedel–Crafts cyclization. Treatment of compounds **16a**–**16d** with Bi(OTf)_3_ (10 mol%) in anhydrous DCM at 30 °C for 2 h afforded the phenanthrene framework (**17a**–**17d**) through regioselective cyclization in 88.4% yield. Finally, the removal of the methyl ester protecting groups was accomplished by basic hydrolysis using aqueous NaOH solution (2.0 M) in a 3:1 (*v*/*v*) methanol/water mixture at room temperature for 12 h, delivering the target carboxylic acids **18a**–**18d** (Scheme 7).

### 3.2. Study on the Toxicity of Denitroaristolochic Acids

This study systematically evaluated the cytotoxic effects of aristolochic acids (AAs) on HK2 cells (the HK2 cell lines were from the Cell Bank of the Chinese Academy of Sciences) using the CCK-8 assay. Quantitative analysis revealed that denitroaristolochic acid III (DAA-III) and denitroaristolochic acid IV (DAA-IV) exhibited a certain degree of cytotoxicity, with half-maximal inhibitory concentration (IC_50_) values of 371 μM and 515 μM, respectively. Although the absence of nitro functional groups significantly reduced the cytotoxic potency of these compounds, their cytotoxic effects were not completely abolished, suggesting that nitro substituents may not constitute an essential structural determinant for AA-induced toxicity in HK2 cells. Mechanistic investigations indicated that, in contrast to the canonical genotoxic pathway of classical AAs involving nitroreductive metabolism to form DNA adducts, denitro derivatives may mediate cellular damage through non-DNA adduct-dependent molecular mechanisms. This finding provides novel insights into the toxicological mechanisms of aristolochic acid analogs, and their precise mode of action warrants further elucidation through integrated toxicological approaches, including metabolomic profiling, signaling pathway screening, and molecular docking studies.

In the toxicity assessment, the CCK-8 assay elucidated distinct cytotoxic profiles of denitroaristolochic acid derivatives toward HK2 cells [10] (Table 4). Quantitative analysis revealed significantly higher IC_50_ values for DAA-III (371 μM) and DAA-IV (515 μM) compared to the prototypical nitro-containing aristolochic acid AA-I (270 μM). This comparative toxicity pattern suggests that while nitro group elimination fails to completely abrogate cellular toxicity, it may attenuate toxicological effects through potential modifications in metabolic activation pathways or alternative molecular targets. Notably, reliable quantification of DAA-II and DAA-V cytotoxicity was precluded by analytical sensitivity limitations (ND), suggesting that their toxicity, if present, falls below the assay’s current detection thresholds. Early structure–activity relationship analysis demonstrated that methoxy substitution exerted negligible influence on cytotoxic potency, whereas nitro group deletion appeared to disrupt the classical toxicity mechanism mediated through DNA adduct formation. This mechanistic shift implies that DAA derivatives might induce cellular damage via non-adduct dependent pathways, potentially involving oxidative stress cascades or mitochondrial dysfunction. These findings challenge the conventional paradigm of aristolochic acid toxicity centered on DNA adduct formation, proposing alternative molecular mechanisms that warrant systematic investigation in subsequent toxicological studies. The differential cytotoxic profiles observed among structural analogs provide critical insights for guiding future structure optimization of aristolochic acid derivatives with improved safety profiles.

## 4. Discussions

Based on retrosynthetic analysis design principles, an efficient synthetic route for the preparation of denitroaristolochic acids II–V series has developed in this study. The key steps include (1) a Br2 bromination strategy affording the brominated intermediate (compound **2**) in 50.8% yield; (2) construction of the crucial boronic ester building block via palladium-catalyzed Miyaura borylation; (3) precise assembly of the biaryl architecture through transition metal-catalyzed Suzuki coupling reaction. The biaryl framework was effectively assembled through Suzuki–Miyaura cross-coupling, followed by precise construction of the phenanthrene core through sequential Wittig reaction and Bi(OTf)_3_-catalyzed Friedel–Crafts cyclization. The cyclization step exhibited exceptional efficiency, as exemplified by compound **17a** with 88.4% isolated yield, and subsequent deprotection provided the corresponding carboxylic acid derivative (compound **18a**) in 83.2% yield.

The synthetic challenges encountered and corresponding optimization strategies presented in this study establish valuable precedents for derivatization research of analogous natural products. Initial synthetic attempts toward intermediate B revealed the limitations of conventional methodologies, as neither Henry reaction nor metal-mediated reductive systems (Zn/HCl or Fe/AcOH) proved effective in achieving the desired transformation. This impasse was resolved through the strategic integration of Darzens condensation with Wittig reaction, which successfully addressed the inherent selectivity challenges. Notably, systematic optimization of carboxylic acid hydrolysis parameters, particularly through solvent screening and precise pH modulation, markedly enhanced reaction efficiency. However, experimental data indicate that specific transformation steps (e.g., decarboxylation of compound **9**) remain constrained by significant yield limitations (<10%), suggesting the need for further investigation into weak acid catalysis combined with thermally stable solvent systems. These findings collectively emphasize the critical importance of modular synthetic design coupled with synergistic condition optimization in the construction of complex natural product architectures.

## 5. Conclusions

Focusing on the structural characteristics of denitroaristolochic acids II–V, this study developed a modular synthetic strategy with three key breakthroughs: (1) optimized bromination conditions significantly improved the preparation efficiency of intermediate 2 (50.8% yield); (2) Suzuki–Miyaura coupling for biaryl framework assembly; (3) innovative use of bismuth(III) triflate-catalyzed cyclization achieved phenanthrene core construction with 88.4% yield, where all compounds were confirmed by comprehensive NMR and LC-MS analyses, demonstrating excellent reproducibility and product purity throughout the synthetic sequence.

The CCK-8 cytotoxicity assessment demonstrated that denitroaristolochic acid derivatives DAA-III (371 μM) and DAA-IV (515 μM) exhibited lower cytotoxicity toward HK2 cells compared to their nitro-containing prototype AA-I (270 μM), suggesting that nitro group elimination, although not completely abolishing toxicity, may attenuate cytotoxic effects possibly through altering metabolic activation pathways or shifting toward non-DNA adduct-dependent mechanisms such as oxidative stress and mitochondrial dysfunction, thereby challenging the conventional DNA adduct-dominant toxicity paradigm and providing novel insights for structural optimization strategies.

This study establishes standardized synthetic protocols and toxicological evaluation systems for denitroaristolochic acid derivatives, confirming the non-essential role of nitro groups in toxicity while proposing future integration of metabolomics and pathway analysis to elucidate toxicity mechanisms and explore pharmacological potential, with synthetic methodologies applicable to other analogues to support toxicity warning systems and structural optimization in traditional medicine development.

## Figures and Tables

**Table 1:**
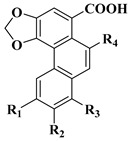
Chemical structures and classification characteristics of major aristolochic acids.

No.	General Name	Abbreviation	Substituents	CAS No.
1	aristolochic acid I (aristolochic acid A)	AA-I	R_1_=R_2_=H, R_3_=OCH_3,_ R_4_=NO_2_	313-67-7
2	aristolochi acid IA	AA-IA	R_1_=R_2_=H, R_3_=OH_,_ R_4_=NO_2_	38965-71-8
3	aristolochi acid II (aristolochi acid B)	AA-II	R_1_=R_2_=R_3_=H_,_ R_4_=NO_2_	475-80-9
4	denitroaristolochic acid II	DAA-II	R_1_=R_2_=R_3_=R_4_=H	70195-23-2
5	aristolochi acid III	AA-III	R_2_=R_3_=H, R_1_=OCH_3,_ R_4_=NO_2_	7267-92-7
6	aristolochi acid IIIA (aristolochi acid C)	AA-IIIA	R_2_=R_3_=H, R_1_=OH_,_ R_4_=NO_2_	4849-90-5
7	denitroaristolochic acid III	DAA-III	R_2_=R_3_=H, R_1_=OCH_3,_ R_4_=H	/
8	aristolochi acid IV	AA-IV	R_2_=H, R_1_=R_3_=OCH_3,_ R_4_=NO_2_	15918-62-4
9	aristolochi acid IVA (aristolochi acid D)	AA-IVA	R_2_=H, R_1_=OH, R_3_=OCH_3,_ R_4_=NO_2_	17413-38-6
10	aristolochi acid IVB	AA-IVB	R_2_=H, R_1_=OCH_3_, R_3_=OH_,_ R_4_=NO_2_	1374601-32-7
11	denitroaristolochic acid IV	DAA-IV	R_2_=R_4_=H, R_1_=R_3_=OCH_3_	64543-59-5
12	aristolochi acid V	AA-V	R_3_=H, R_1_=R_2_=OCH_3,_ R_4_=NO_2_	108766-28-5
13	aristolochi acid VA	AA-VA	R_3_=H, R_1_=OH, R_2_=OCH_3,_ R_4_=NO_2_	108779-46-0
14	denitroaristolochic acid V	DAA-V	R_3_=R_4_=H, R_1_=R_2_=OCH_3_	/

**Table 2:** Optimization of reaction conditions for compound **2**.

Entry	Reagent	Catalyst	Solvent	Time	Temperature	Yield
1	NBS (1.1 eq)	*m*-CPBA (0.1 eq)	methanol	12 h	r.t./65 °C	—
2	NBS (1.1 eq)	*m*-CPBA (0.1 eq)	DCM	12 h	r.t./40 °C	—
3	NBS (1.1 eq)	*m*-CPBA (0.1 eq)	THF	12 h	r.t./66 °C	—
4	dibromohydantoin (1.1 eq)	*m*-CPBA (0.1 eq)	methanol	12 h	r.t./65 °C	—
5	dibromohydantoin (1.1 eq)	*m*-CPBA (0.1 eq)	DCM	12 h	r.t./65 °C	—
6	dibromohydantoin (1.1 eq)	*m*-CPBA (0.1 eq)	THF	12 h	r.t./40 °C	—
7	dibromohydantoin (1.1 eq)	/	acetic acid	12 h	r.t.	—
8	NBS (1.1 eq)	/	acetic acid	12 h	r.t.	—
9	Br_2_ (1.1 eq)	/	acetic acid	6 h	r.t.	50.8%

**Table 3:** Optimization of reaction conditions for carboxyl group hydrolysis.

Entry	Reagent	pH	Solvent	Time	Temperature	Yield
1	H_2_SO_4_	0.5	THF/DCM	overnight	Reflux	<10%
2	H_2_SO_4_	1.5	THF/DCM	overnight	Reflux	<10%
3	H_2_SO_4_	2.5	THF/DCM	overnight	Reflux	<10%
4	HCl	0.5	THF/DCM	overnight	Reflux	<10%
5	HCl	1.5	THF/DCM	overnight	Reflux	<10%
6	HCl	2.5	THF/DCM	overnight	Reflux	<10%
7	H_3_PO_4_	0.5	THF/DCM	overnight	Reflux	<10%
8	H_3_PO_4_	1.5	THF/DCM	overnight	Reflux	<10%
9	H_3_PO_4_	2.5	THF/DCM	overnight	Reflux	<10%
10	H_3_PO_4_	1.5	diphenyl ether	1 h	260 °C	——

**Table 4:** Cytotoxic effects of the target compounds on HK2 cells in vitro.

No.	Compounds	Abbreviation	IC_50_ (μM) *
1	denitroaristolochic acid II	DAA-II	ND
2	denitroaristolochic acid III	DAA-III	371
3	denitroaristolochic acid IV	DAA-IV	515
4	denitroaristolochic acid V	DAA-V	ND
5	aristolochic acid I	AA-I	270

* ND: not detected.

## Data Availability

The original contributions presented in this study are included in the article/Supplementary Material; further inquiries can be directed to the corresponding authors.

## Supplementary Materials

The following supporting information can be downloaded at https://www.mdpi.com/article/10.3390/biom15071014/s1. The ^1^H-NMR, ^13^C-NMR and HRMS of the target compounds and key intermediates.

## Abbreviations

The following abbreviations are used in this manuscript:

AA	aristolochic acid
B_2_pin_2_	bis(pinacolato)diboron
Bi(OTf)_3_	bismuth triflate
CCK-8	cell counting kit-8
DAA	denitroaristolochic acid
DCM	dichloromethane
DME	dimethoxyethane
DMEM	dulbecco’s modified eagle medium
DMF	*N*,*N*-dimethylformamide
DMSO	dimethyl sulfoxide
ESI	electrospray ionization
HK2	human renal cortical proximal tubular epithelial cell
HPLC	high performance liquid chromatography
HRMS	high-resolution mass spectrometry
IARC	International Agency for Research on Cancer
LC-MS	liquid chromatography–mass spectrometry
LD_50_	median lethal dose
*m*-CPBA	*m*-chloroperbenzoic acid
MMTPCl	methoxymethyltriphenylphosphonium chloride
NBS	*N*-bromosuccinimide
ND	not detected
NMR	nuclear magnetic resonance
Pd(dppf)Cl_2_	dichloro [1,1′-bis(diphenylphosphino)ferrocene]palladium(II)
Ph(PPh_3_)_4_	tetrakis(triphenylphosphine)palladium
r.t.	room temperature
SAR	structure–activity relationships
t-BuOK	potassium tert-butoxide
t-BuONa	sodium tert-butoxide
TEA	triethylamine
THF	tetrahydrofuran
TLC	thin layer chromatography
TMS	tetramethylsilane

## Appendix A

The chemical names, chemical structures, and CAS registry numbers of target compounds and key intermediates are listed as follows:

**No.**	**Chemical Name**	**Chemical Structure**	**CAS No.**
compound **2**	3-bromo-4,5-dihydroxybenzaldehyde		16414-34-9
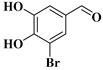
compound **3**	7-bromobenzo[*d*][1,3]dioxole-5-carbaldehyde		19522-96-4
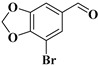
compound **4**	methyl 7-bromobenzo[*d*][1,3]dioxole-5-carboxylate		103095-80-3
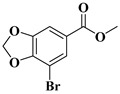
compound **6**	(*E*)-1-bromo-3,5-dimethoxy-2-(2-nitrovinyl)benzene		2229645-24-1
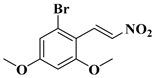
compound **13a**	2-(4,4,5,5-tetramethyl-1,3,2-dioxaborolan-2-yl)benzaldehyde		380151-85-9
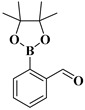
compound **13b**	4-methoxy-2-(4,4,5,5-tetramethyl-1,3,2-dioxaborolan-2-yl)benzaldehyde		1196474-59-5
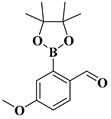
compound **13c**	2,4-dimethoxy-6-(4,4,5,5-tetramethyl-1,3,2-dioxaborolan-2-yl)benzaldehyde		1265360-45-9
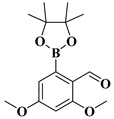
compound **13d**	4,5-dimethoxy-2-(4,4,5,5-tetramethyl-1,3,2-dioxaborolan-2-yl)benzaldehyde		1098755-60-2
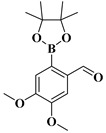
compound **17a**	methyl phenanthro [3,4-d][1,3]dioxole-5-carboxylate		310396-78-2
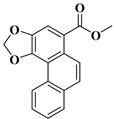
compound **17c**	methyl 8,10-dimethoxyphenanthro [3,4-d][1,3]dioxole-5-carboxylate		64543-60-8
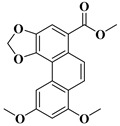
compound **18a**	phenanthro [3,4-d][1,3]dioxole-5-carboxylic acid		70195-23-2
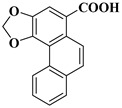
compound **18c**	8,10-dimethoxyphenanthro [3,4-d][1,3]dioxole-5-carboxylic acid		64543-59-5
