# Supplementary material for: Synthesis and Cytotoxicity Evaluation of Denitroaristolochic Acids: Structural Insights and Mechanistic Implications in Nephrotoxicity
Source: Biomolecules. 2025 Jul 14;15(7):1014. doi: 10.3390/biom15071014 (PMC12293755; doi:10.3390/biom15071014)

biomolecules

Supplementary Materials

Synthesis and Cytotoxicity Evaluation of Denitroaristolochic Acids: Structural Insights and Mechanismic Implications in Nephrotoxicity

Jianfei Gao [1], Mengtong Zhao [1], Jianhua Su [1], Yi Gao [1], Xiaofeng Zhang [1], Yongzhao Ding [1], Xiaoping Liu [1,*], Yang Luan [2,*] and Chun Hu [2,*]

[1] Key Laboratory of Structure-based Drug Design & Discovery (Ministry of Education), Shenyang Pharmaceutical University, Shenyang110016, China; gaojianfei1996@163.com(J. G.); zmt18240302933@163.com (M. Z.); 18522280724@163.com (J. S.); yigao2022@163.com (Y. G.); z1121628369@126.com (X. Z.); iyuzuru@126.com (Y. D.); xiaopingliu@syphu.edu.cn (X. L.) and chunhu@syphu.edu.cn (C. H.)

[2] School of Public Health, Hongqiao International Institute of Medicine, Shanghai Jiao Tong University School of Medicine, Shanghai 200025, China; yluan@sjtu.edu.cn (Y. L.)

* Correspondences: yluan@sjtu.edu.cn (Y. L.); chunhu@syphu.edu.cn (C. H.); Tel.: +86-24-43520246 (C. H.)

^{1}H NMR spectra of compound 2

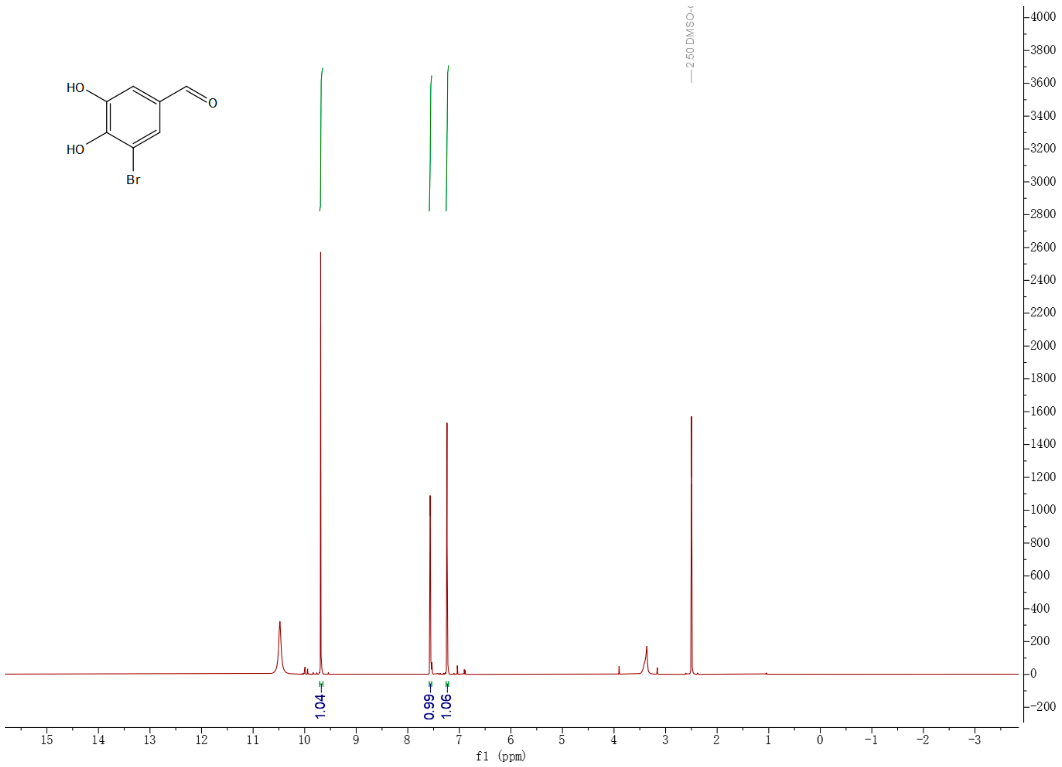

^{1}H NMR spectra of compound 3

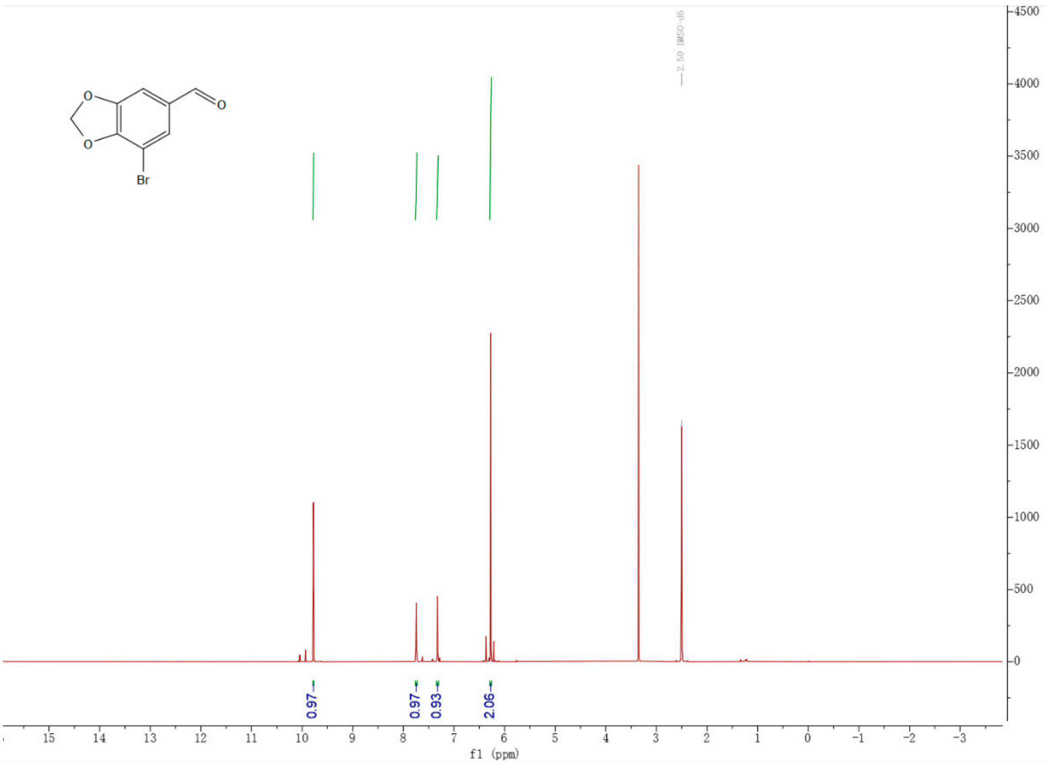

^{1}H NMR spectra of compound 4

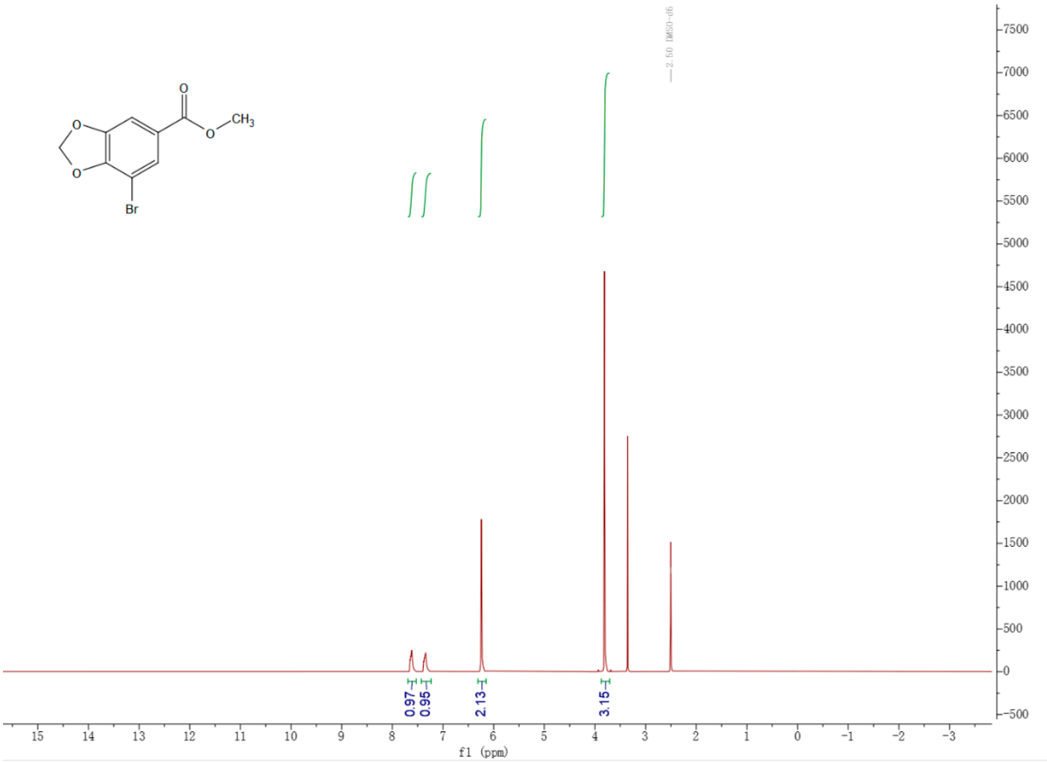

^{1}H NMR spectra of compound 14a

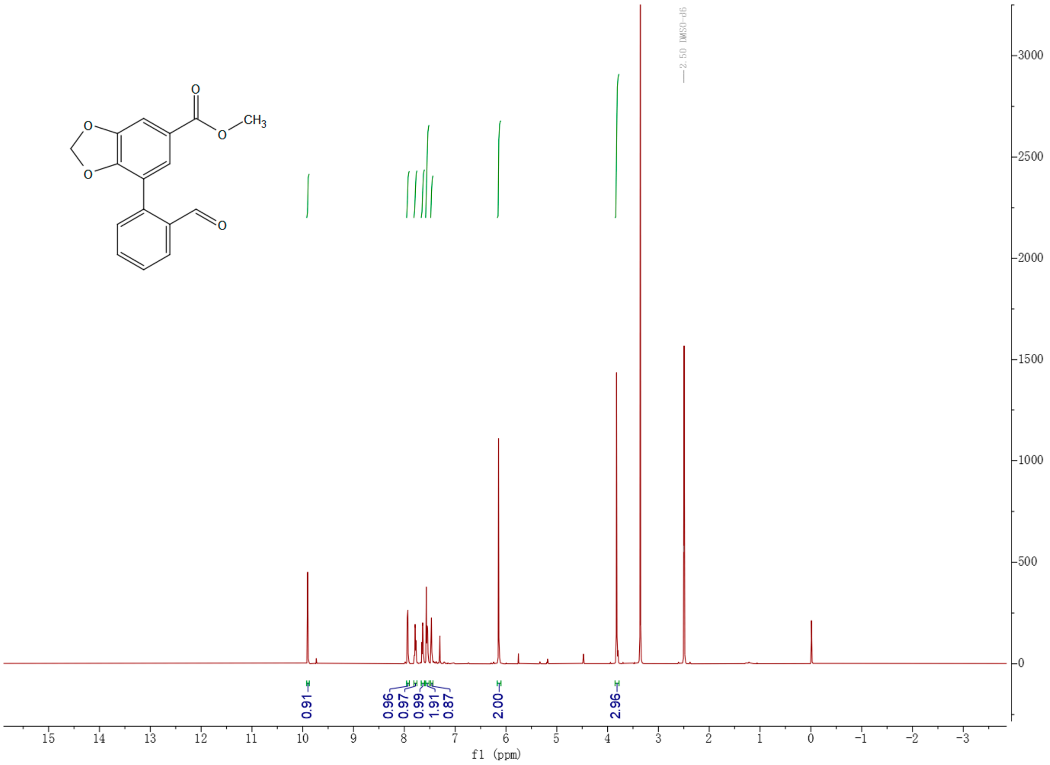

^{1}H NMR spectra of compound 14b

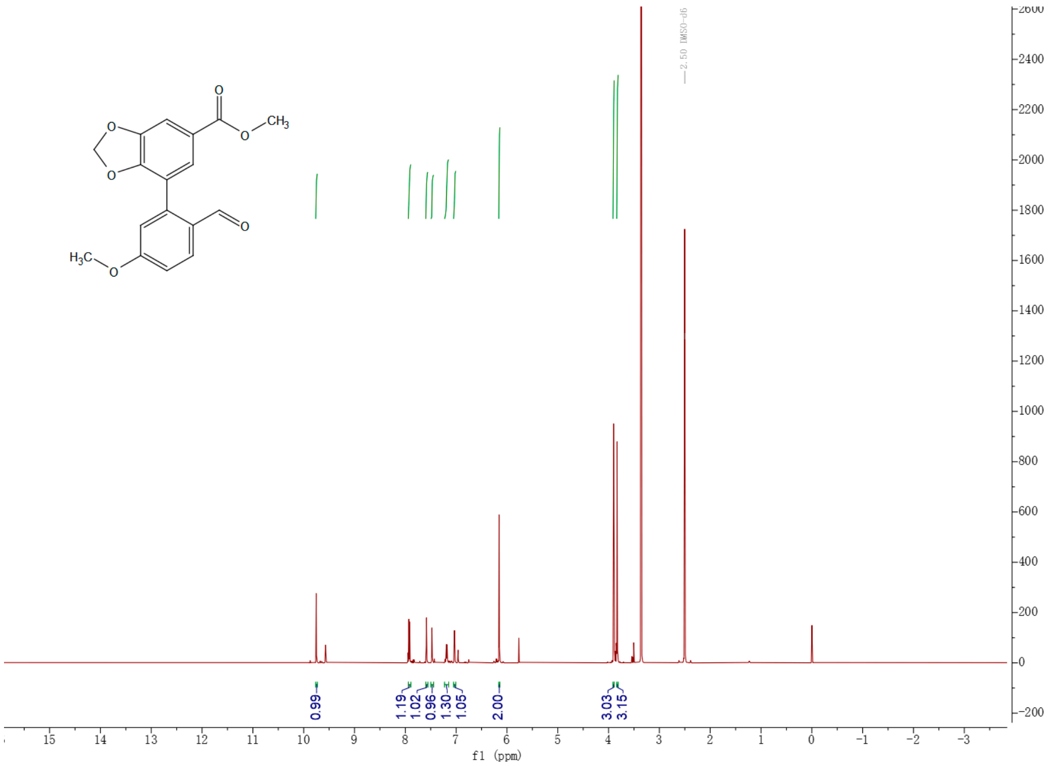

^{1}H NMR spectra of compound 14c

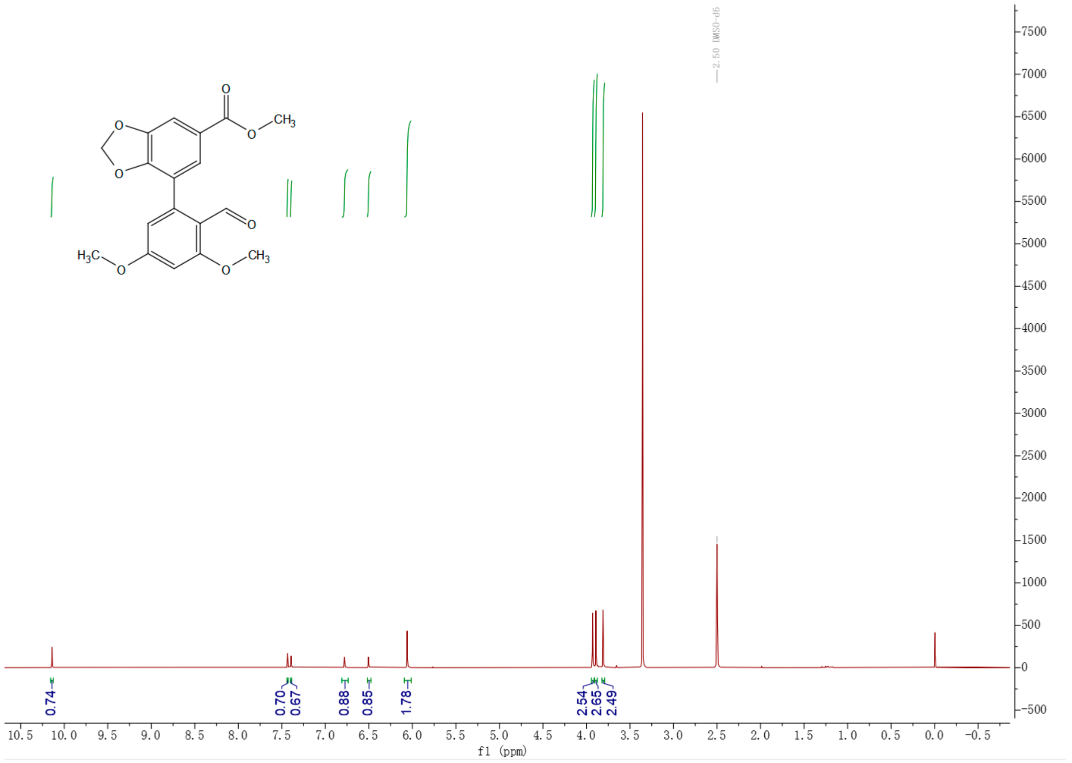

^{1}H NMR spectra of compound 14d

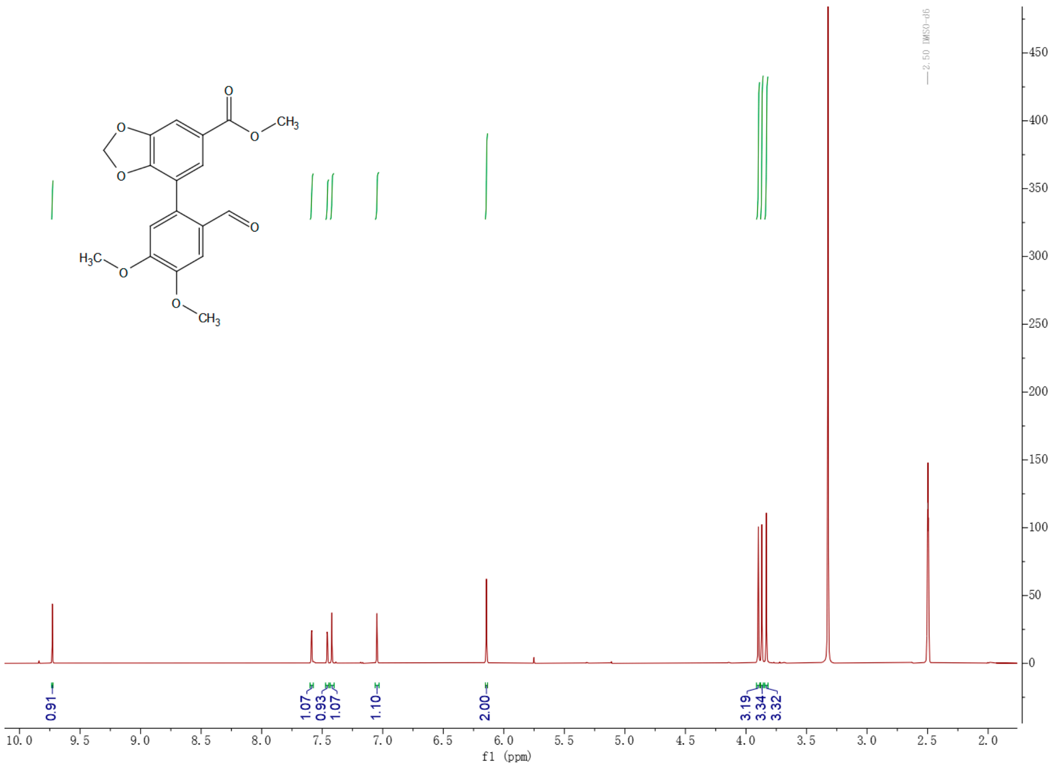

^{1}H NMR spectra of compound 16a

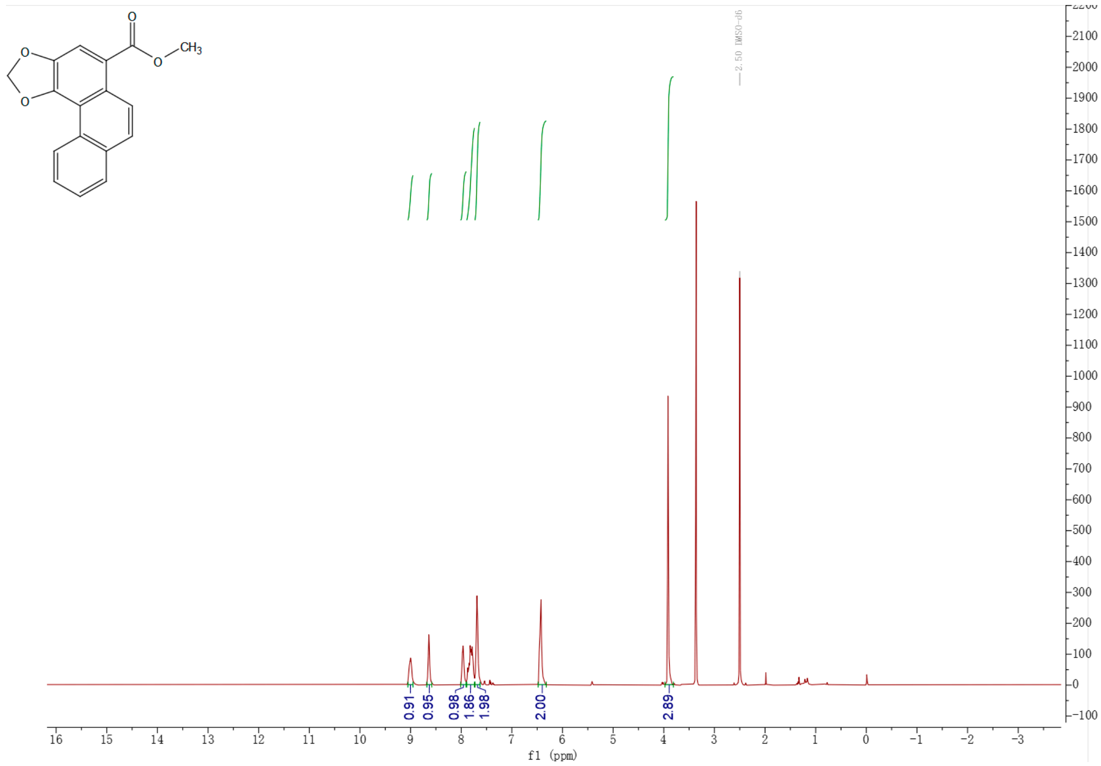

^{1}H NMR spectra of compound 16b

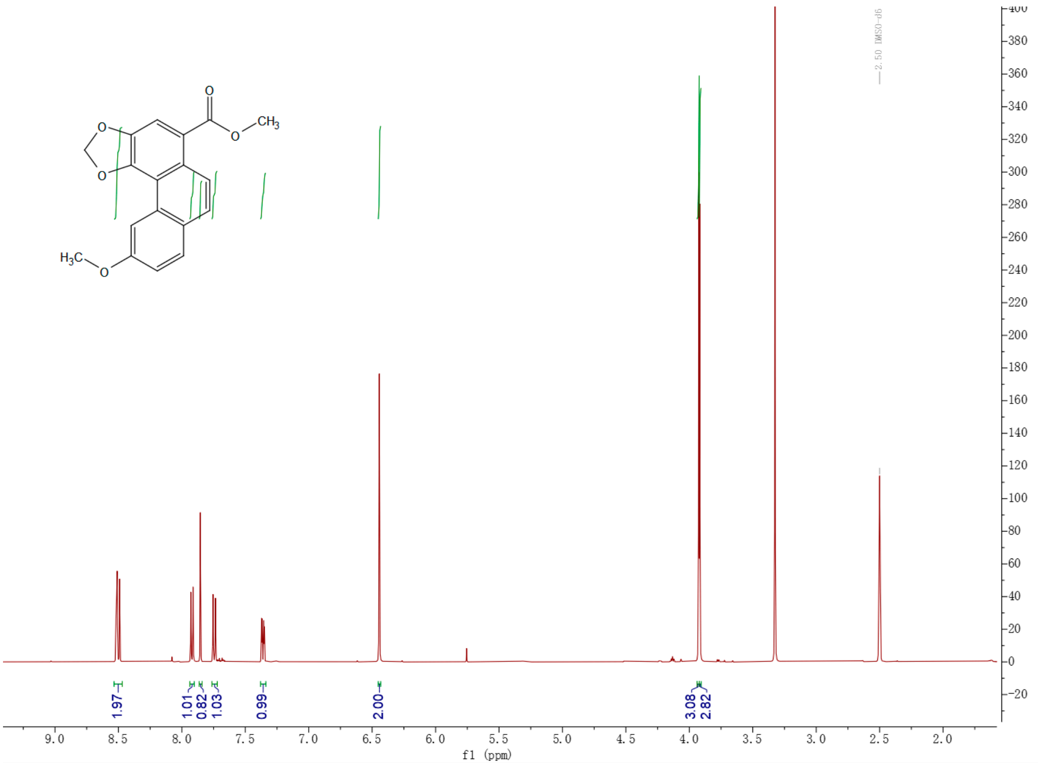

^{1}H NMR spectra of compound 16c

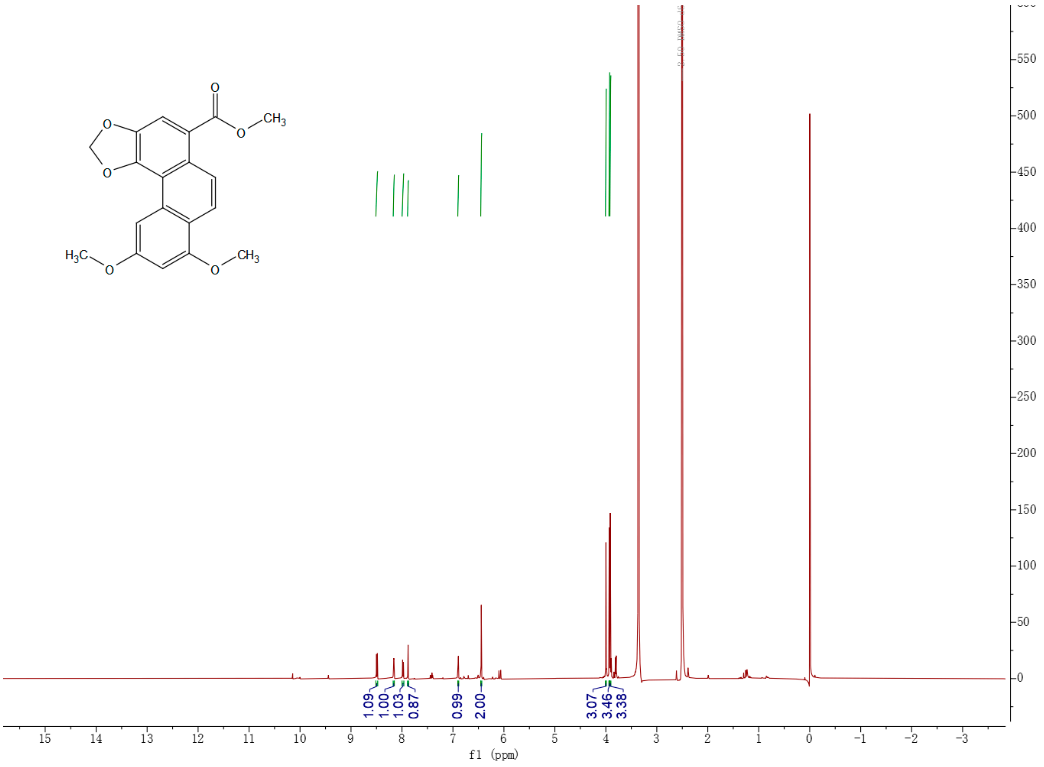

^{1}H NMR spectra of compound 16d

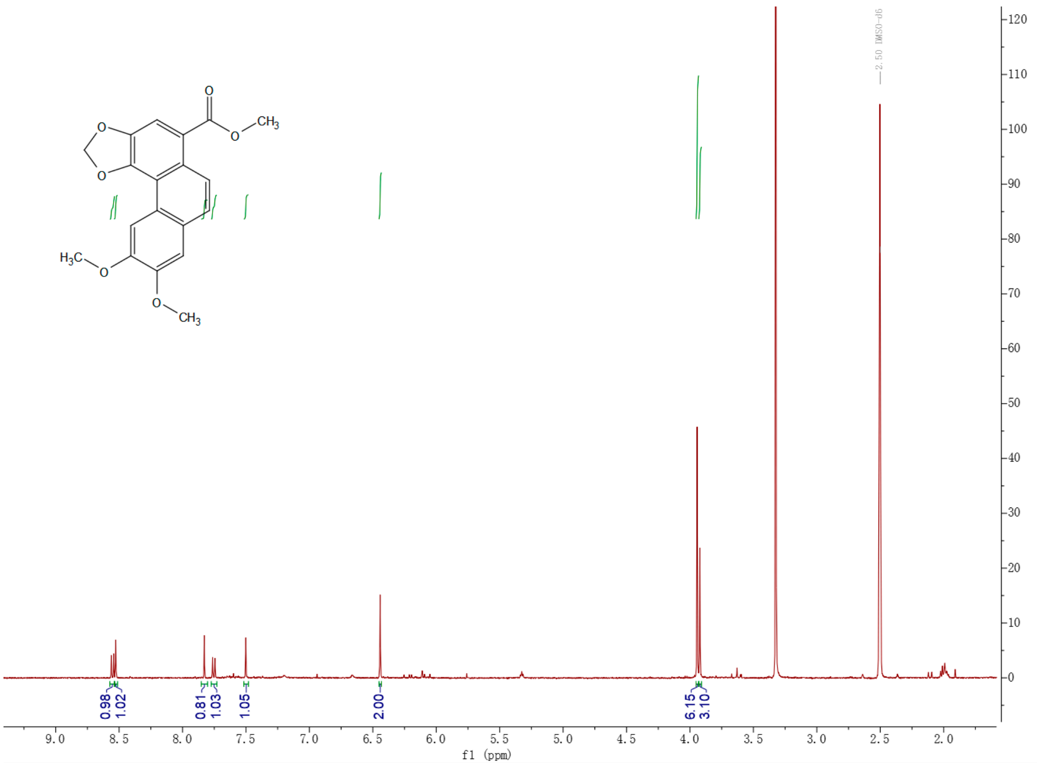

^{1}H NMR spectra of compound 17a

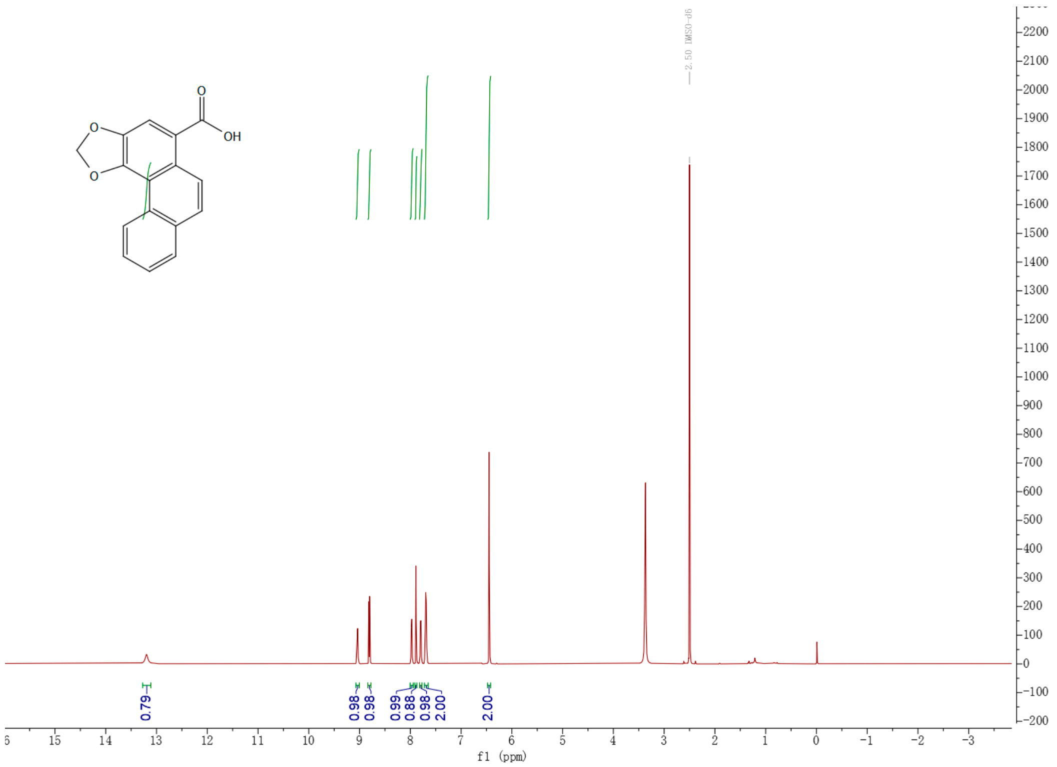

^{13}C NMR spectra of compound 17a

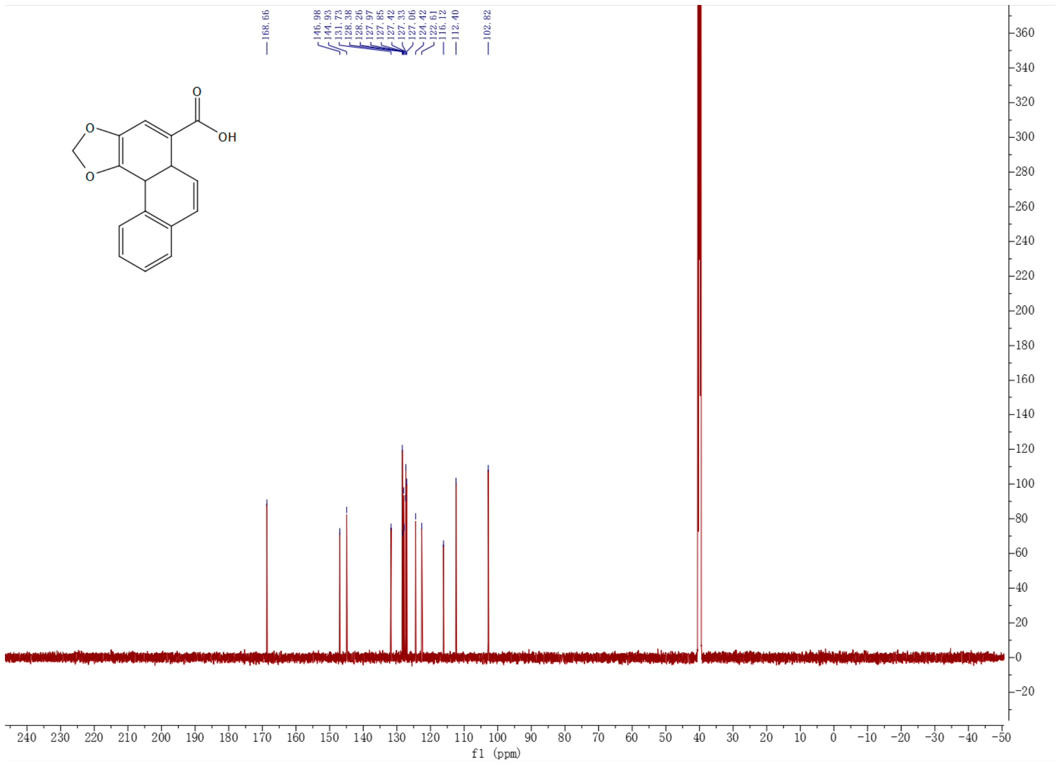

ESI-HRMS spectra of compound 17a

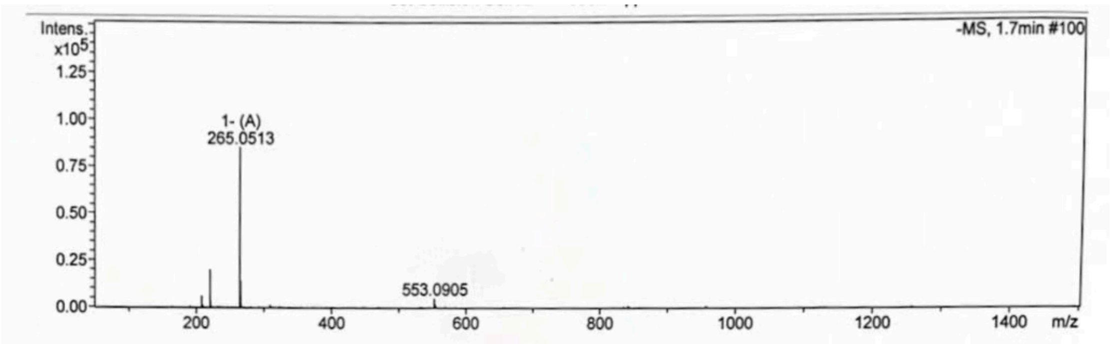

^{1}H NMR spectra of compound 17b

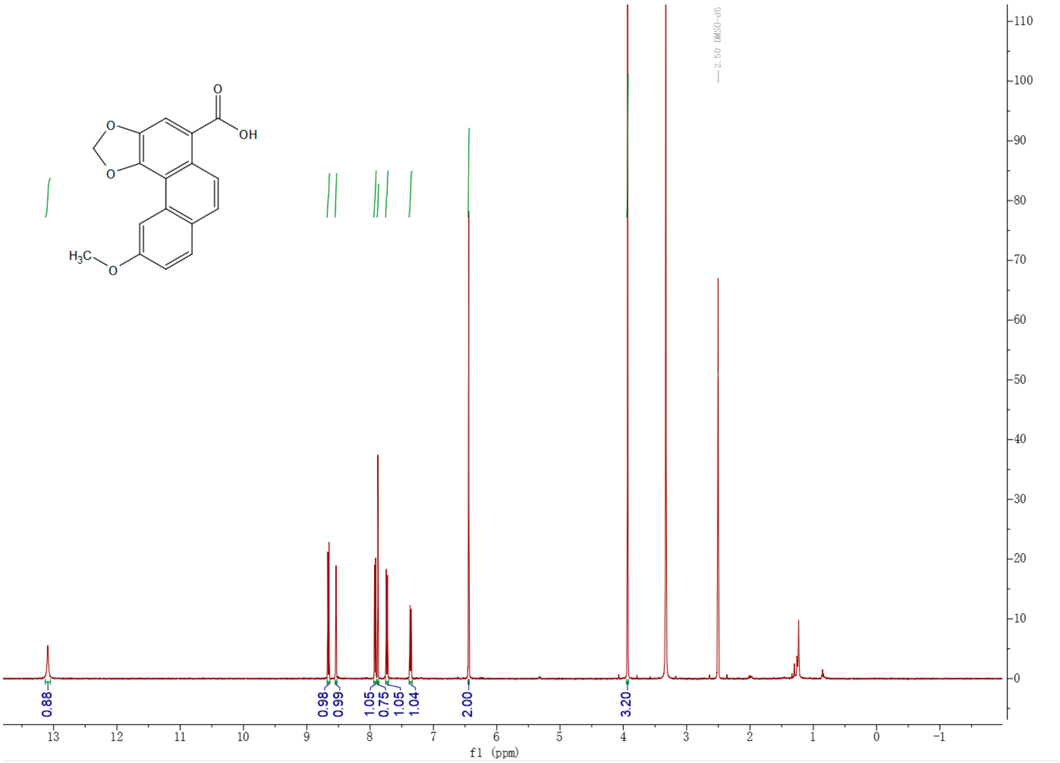

^{13}C NMR spectra of compound 17b

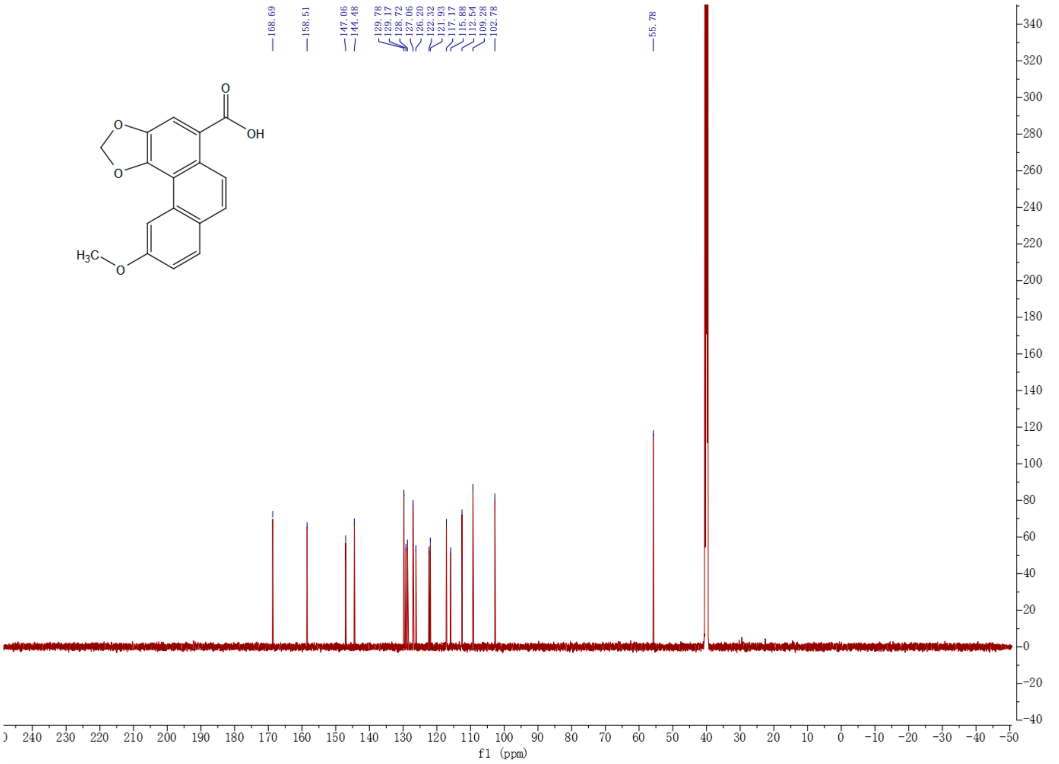

ESI-HRMS spectra of compound 17b

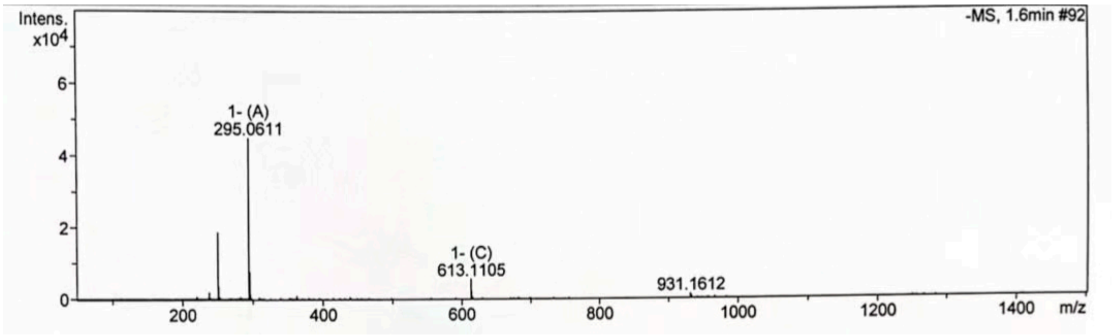

^{1}H NMR spectra of compound 17c

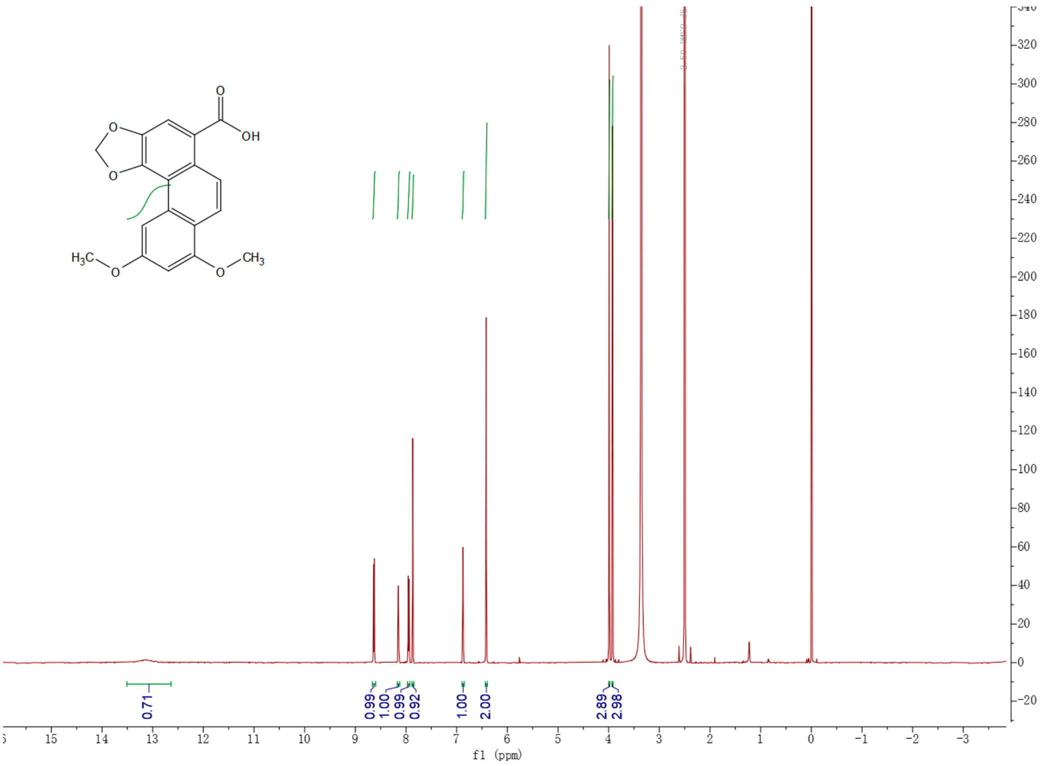

^{13}C NMR spectra of compound 17c

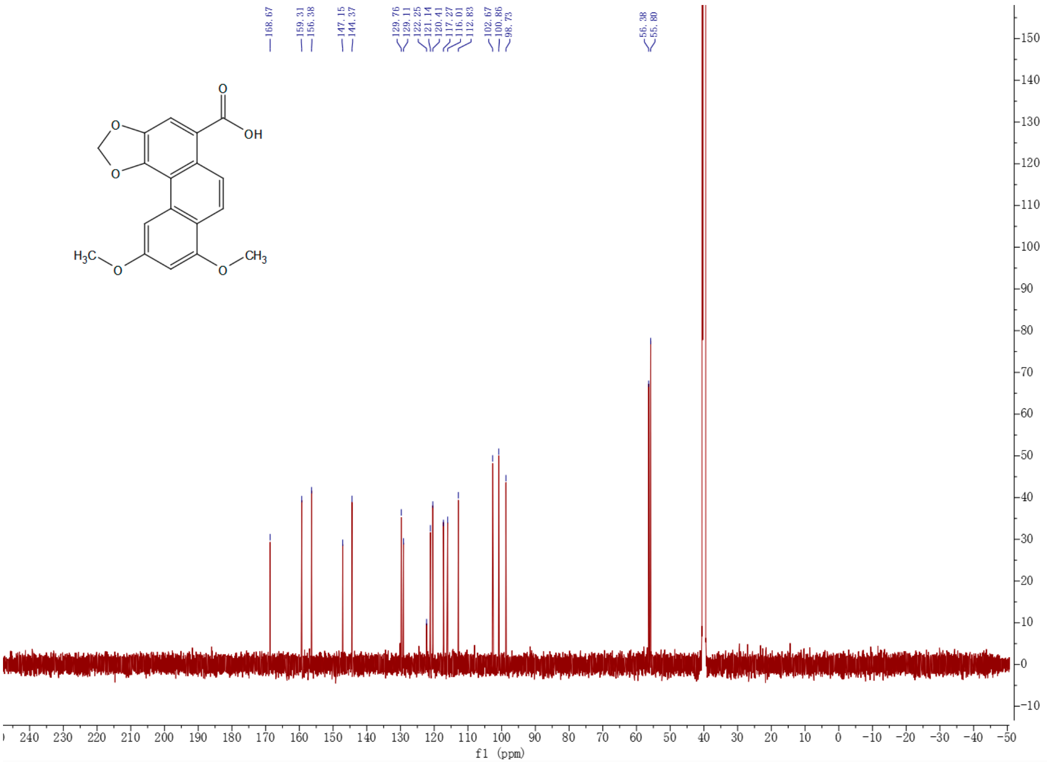

ESI-HRMS spectra of compound 17c

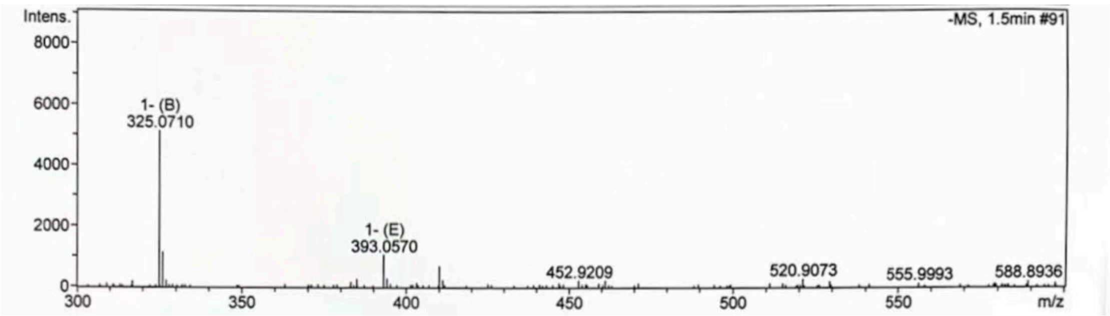

^{1}H NMR spectra of compound 17d

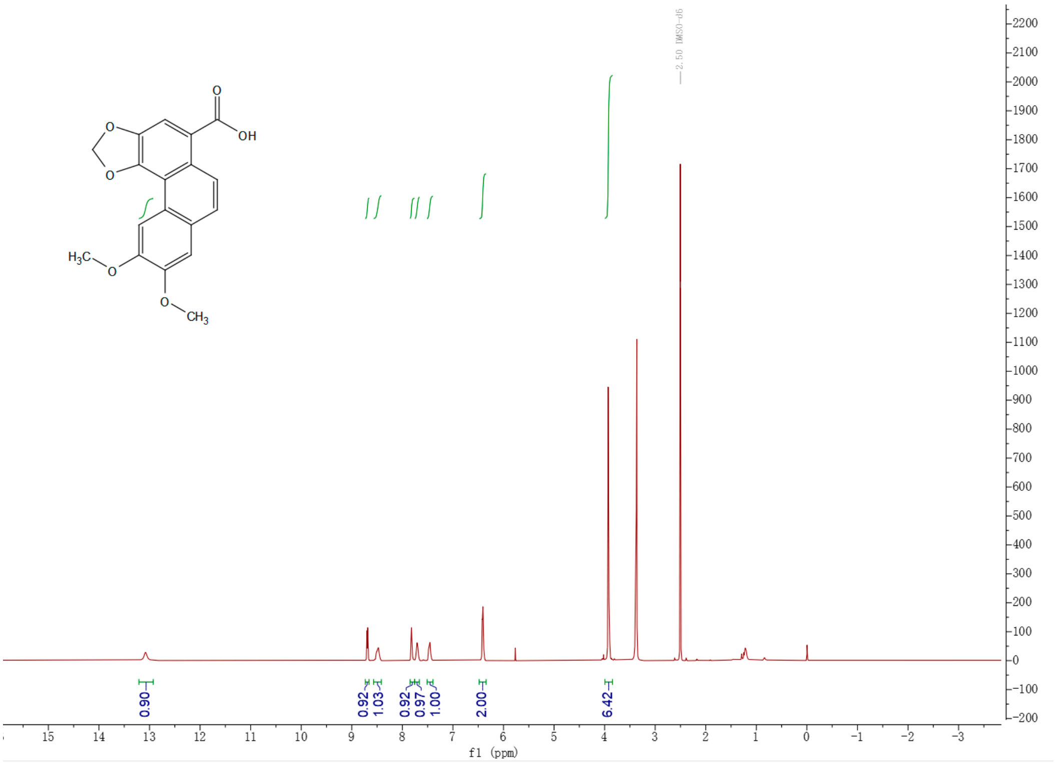

^{13}C NMR spectra of compound 17d

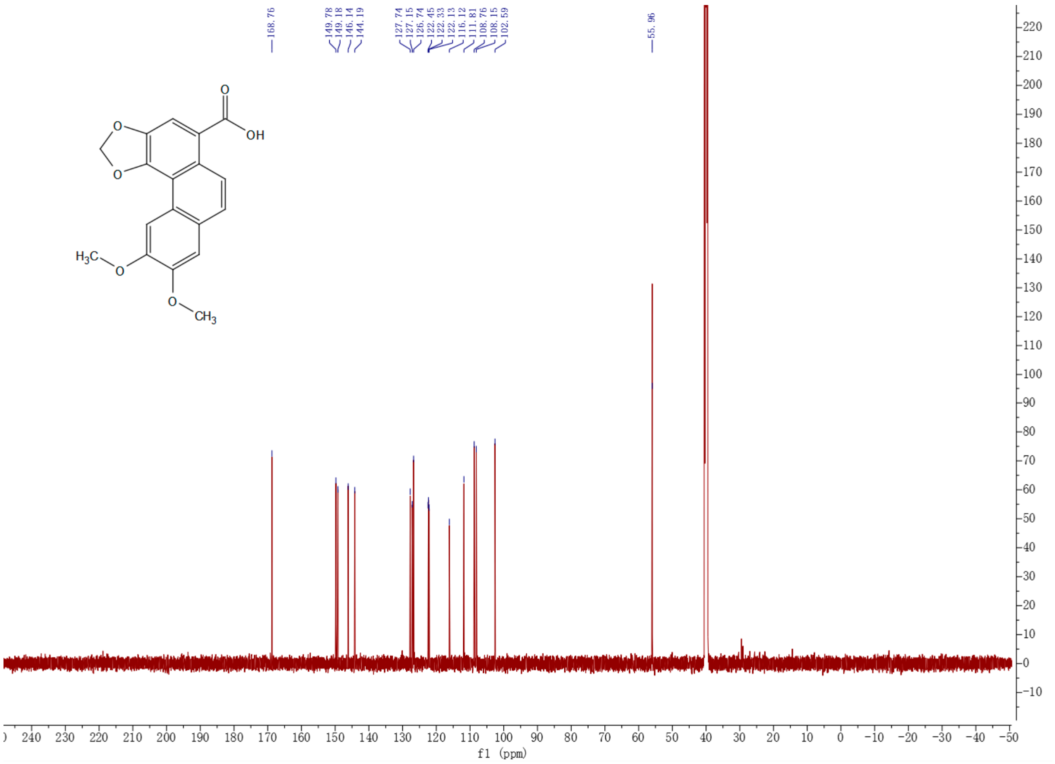

ESI-HRMS spectra of compound 17d

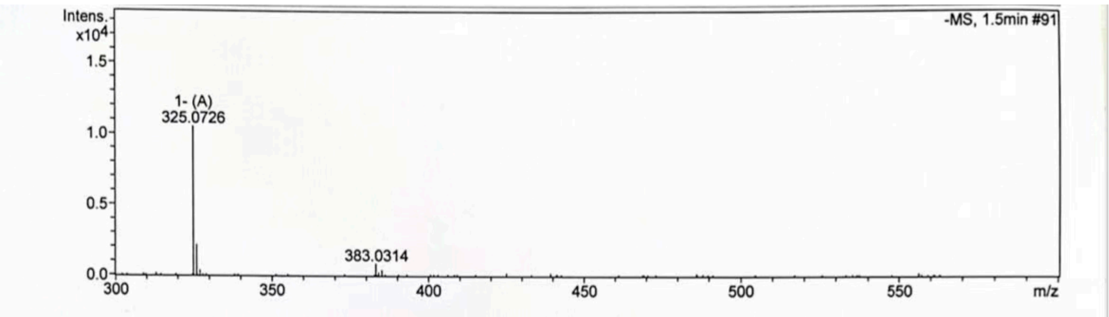

Supplement: Supplementary file 1 [file biomolecules-15-01014-s001.zip › biomolecules-3679258-supplementary.pdf]
